# miR-1306 Mediates the Feedback Regulation of the TGF-β/SMAD Signaling Pathway in Granulosa Cells

**DOI:** 10.3390/cells8040298

**Published:** 2019-03-31

**Authors:** Liu Yang, Xing Du, Lu Liu, Qiuyu Cao, Zengxiang Pan, Qifa Li

**Affiliations:** College of Animal Science and Technology, Nanjing Agricultural University, Nanjing 210095, China; 2016105012@njau.edu.cn (L.Y.); duxing@njau.edu.cn (X.D.); 18994091721@163.com (L.L.); 13675138768@163.com (Q.C.); owwa@njau.edu.cn (Z.P.)

**Keywords:** miR-1306, TGF-β/SMAD signaling pathway, TGFBR2, granulosa cell apoptosis

## Abstract

Transforming growth factor-β receptor II (TGFBR2), the type II receptor of the TGF-β/SMA- and MAD-related protein (SMAD) signaling pathway, plays a crucial role in TGF-β signal transduction and is regulated by multiple factors. Nevertheless, the modulation of the non-coding RNA involved in the process of *TGFBR2* expression in ovaries is not well studied. In our study, we isolated and characterized the 3′-untranslated region (UTR) of the porcine *TGFBR2* gene and microRNA-1306 (miR-1306) was identified as the functional miRNA that targets TGFBR2 in porcine granulosa cells (GCs). Functional analysis showed that miR-1306 promotes apoptosis of GCs as well as attenuating the TGF-β/SMAD signaling pathway targeting and impairing TGFBR2 in GCs. Moreover, we identified the miR-1306 core promoter and found three potential SMAD4-binding elements (SBEs). Luciferase and chromatin immunoprecipitation (ChIP) assays revealed that the transcription factor SMAD4 directly binds to the miR-1306 core promoter and inhibits its transcriptional activity. Furthermore, the TGF-β/SMAD signaling pathway is modulated by SMAD4 positive feedback via inhibition of miR-1306 expression in GCs. Collectively, our findings provide evidence of an epigenetic mechanism that modulates as well as mediates the feedback regulation of the classical TGF-β/SMAD signaling pathway in GCs from porcine ovaries.

## 1. Introduction

The transforming growth factor beta (TGF-β)/SMA- and MAD-related protein (SMAD) signaling pathway plays a significant role in regulating numerous processes in the cell, including cellular proliferation [1], differentiation [2], and the cell cycle [3], as well as apoptosis [4], by introducing extracellular signal transduces into the cell nucleus. The classic TGF-β/SMAD signaling pathway consists of a ligand (TGF-β1) and two transmembrane serine-threonine kinase receptors, namely TGF-β receptor I (TGFBR1 or ALK5) and TGF-β receptor II (TGFBR2), as well as SMADs (SMAD2/3/4). Activation of the signal depends on the interaction between the ligand and TGFBR2 leading to the dimerization of TGFBR2 and TGFBR1. During this process, TGFBR1 is phosphorylated and activated by the kinase activity of TGFBR2. Activated TGFBR1 phosphorylates downstream molecules SMAD2 and SMAD3, subsequently leading to the formation of a trimeric complex with SMAD4. Finally, this complex translocates into the nucleus and modulates downstream gene transcription in response to extracellular signals [5]. Therefore, as a core transmembrane receptor, TGFBR2 plays a significant role in the TGF-β signal transduction. For example, odontoblast-specific TGFBR2 conditional knockout in mice results in the loss of responsiveness to TGF-β along with inactivation of the TGF-β/SMAD pathway, causing impaired matrix formation and pulpal obliteration in odontoblasts [6]. The mutational inactivation of *TGFBR2* can inhibit TGF-β/SMAD signaling pathway activity and its tumor-suppressing ability, thereby promoting colon cancer [7]. Besides, down-regulation of *TGFBR2* by using TGFBR2-specific small interfering RNAs (siRNAs) or a small-molecule inhibitor ablates the TGF-β signal-mediated process [8,9].

The expression of *TGFBR2*, in vivo or in vitro, is controlled by multiple factors, including genetic and epigenetic factors [10,11,12,13]. At the genetic level, for instance, the TGFBR2 protein was truncated and inactivated because of *TGFBR2* mutations, thus inhibiting its growth regulatory functions in microsatellite instability-high (MSI-H) cancers [14]. Besides, TGFBR2 is also known to be regulated by several transcription factors such as zinc finger protein 32 (ZNF32) [15] and/or co-activators such as yes-associated protein 1 (YAP-1) [16]. At the epigenetic level, *TGFBR2* expression is widely regulated by various factors, such as microRNAs (miRNAs) and long non-coding RNAs (lncRNAs) [17,18]. For example, miR-93 down-regulates its target gene, *TGFBR2*, in prostate cancer and leads to acceleration of cell growth and invasion [19]. miRNA-520f and miR-7 are known to suppress *TGFBR2* and inhibit epithelial–mesenchymal transition of epithelial cells [20,21]. Furthermore, lncRNAs usually regulate *TGFBR2* expression by interacting with miRNAs. A recent study reported that lnc-small nucleolar RNA host gene 1 (lnc-SNHG1), an overexpressed lncRNA found in the tumor tissues and cells lines originating from invasive pituitary cancer, directly binds to miR-302/372/373/520 and promotes expression of their common target, *TGFBR* [22].

In our previous study, we demonstrated that miR-425 interacts with the classical TGF-β/SMAD signaling pathway by directly targeting *TGFBR2* and suppressing its expression in granulosa cells (GCs) of the porcine ovary [9]. However, further investigation was required to understand the regulatory role of miRNAs in *TGFBR2* expression in GCs. In this study, we isolated and characterized the 3′-untranslated region (UTR) of the porcine *TGFBR2* gene and further identified the potential miRNAs that target *TGFBR2* in GCs. Our findings provide additional evidence of epigenetic mechanisms that regulate the TGF-β signaling pathway in GCs.

## 2. Materials and Methods

### 2.1. Reagents

Porcine TGF-β1 was obtained from R&D Systems (Minneapolis, MN, USA). SMAD4-siRNA, TGFBR2-siRNA, miR-1306 mimics, miR-1306 inhibitors, and their negative control (NC) (NC-siRNA, mimic NC and inhibitor NC) were synthesized and purified by GenePharma (Shanghai, China) (Supplementary Table S1). Antibodies against phospho-SMAD3 (p-SMAD3) (Catalog #D155153-0025), TGFBR2 (Catalog #D151818-0025), SMAD3 (Catalog #D155234-0100), glyceraldehyde-3-phosphate dehydrogenase (GAPDH; Catalog #D198662-0100), horseradish peroxidase (HRP)-conjugated mouse anti-rabbit (Catalog #D110065-0100), and HRP-conjugated goat anti-mouse immunoglobulin G (IgG) (Catalog # 110087-0100) were purchased from Sangon Biotech (Shanghai, China). Dulbecco’s minimum essential medium/nutrient F-12 (DMEM/F-12), fetal bovine serum (FBS), and Opti-MEM were purchased from Gibco (USA). Phosphate-buffered saline (PBS) was obtained from HyClone (USA). Lipofectamine 2000 was obtained from Invitrogen (Carlsbad, CA, USA). The protease and phosphatase inhibitors were purchased from Roche (Basel, Switzerland).

### 2.2. Cell Culture and Transfection

Fresh ovaries were procured from mature sows, which reached the laboratory within 1 h of collection. GCs were harvested from suitable follicles using a procedure described previously [23]. These GCs were seeded into 6-well or 12-well plates and cultured with DMEM/F-12 supplied with 15% FBS in a humidified atmosphere at 37 °C with 5% CO_2_. After 12 h of culture, the porcine GCs were transfected with the appropriate plasmids or oligos using Lipofectamine 2000 and Opti-MEM according to the manufacturer’s protocol. All animal-related experiments were approved by the Animal Ethics Committee at Nanjing Agricultural University, China.

### 2.3. Rapid Amplification of Complementary DNA (cDNA) Ends (RACE)

RACE was done to obtain the full-length *TGFBR2* 3′-UTR according to the instructions provided in the SMARTer^®^ RACE 5′/3′ Kit (TaKaRa, Beijing, China). We introduced a “smart oligo” at the 3′-ends of the reverse-transcribed cDNAs to prepare the 3′ RACE library. The gene-specific oligonucleotide (GSP) AAGGGCGCTTTGCCGAGGTCTATAA was used to amplify the 3′-end of the *TGFBR2* gene from the 3′ RACE library. The products were analyzed using electrophoresis with 1.5% agarose gel and purified by DNA gel Extraction kit (TsingKe, Beijing, China). Purified *TGFBR2* 3′-UTR fragment from 3′ RACE assay was cloned into the pClone007 Blunt Vector (TsingKe, Beijing, China) and verified by sequencing.

### 2.4. Bioinformatic Analysis

The putative MRE of *TGFBR2* 3′-UTR was predicted by RNA hybrid. miRBase was used to obtain the pre- and mature miRNA sequences. miRWalk 2.0 and TargetScan v7.1 was used to predict the miR-1306 targets. Promoter 2.0 Prediction Server was used to predict the promoter of the porcine miR-1306. The binding sites for transcription factors of miR-1306 was predicted by JASPAR server.

### 2.5. RNA Isolation and Quantitative RT-PCR (qRT-PCR)

Total RNA was isolated from follicles and cultured GCs using TRIzol reagent (Invitrogen, USA), and then reverse-transcribed to cDNA using the PrimeScript™ RT Master Mix (Perfect Real Time) (Takara, Beijing, China) according to the manufacturer’s protocol. qRT-PCR was performed in triplicate with AceQ qPCR SYBR Green Master Mix (Takara, Beijing, China) using the ABI Step One system (Applied Biosystems, Foster City, CA, USA) according to the manufacture’s instruction. The mRNA levels of TGFBR2 and DiGeorge Syndrome Chromosomal Region 8 (DGCR8) were normalized to glyceraldehyde-3-phosphate dehydrogenase (GAPDH) and U6 small nuclear RNA (U6) was used as the internal control for miRNA. The 2^−ΔΔCT^ method was used to normalize the relative levels of the target genes. The details of all the primers for qRT-PCR used in this study are provided in Supplementary Table S2.

### 2.6. Western Blotting

Porcine GCs were lysed in radioimmunoprecipitation assay (RIPA) (Bioworld, Nanjing, China) containing 1% phosphatase inhibitor (*v*/*v*). The protein concentration was determined by the BCA Protein Assay Kit (Beyotime, Shanghai, China) and diluted to the same concentration using the 5 × Protein Loading Dye (Sangon, Shanghai, China). Total protein extracts were separated on using SDS-PAGE on 12% gels and electrophoresed for 1 h. Following that, the total protein was transferred onto a PVDF membrane (Millipore, Billerica, MA, USA) and the membrane was blocked with 5% non-fat milk for 2 h. After washing with tris buffered saline tween (TBST) for 15 s, the membrane was incubated with primary antibodies (1:1000 dilution) at 4 °C for 12 h. After that, the membrane was washed thrice for 10 min each time using TBST and incubated with the appropriate secondary antibodies (1:2000 dilution). Chemiluminescence was detected by WesternBright^TM^ BCL (Advansta, Menlo Park, CA USA).

### 2.7. Apoptosis Analysis

48 h after transfection, GCs were harvested, and a cell-counting machine (Becton Dickinson, USA) was used for detection of apoptotic cells based on the principle of fluorescence-activated cell sorting (FACS). According to the manufacturer’s protocol (Vazyme, Shanghai, China), the apoptosis rate was detected by flow cytometry with the fluorescein isothiocyante (FITC) and propidium iodide (PI) signals. The FlowJo v7.6 software (Stanford University, Stanford, CA, USA) was used to analyze the results.

### 2.8. Plasmid Construction

We had previously constructed the overexpression plasmids (pcDNA™3.1 (pcDNA3.1)-SMAD4 and pcDNA3.1-TGFBR2) [9,24]. The different fragments of miR-1306 promoter were amplified and cloned into NheI and SacI sites in pGL-3 reporter vector (Promega). The mutant plasmids were generated by TreliefTM SoSoo Cloning Kit (TsingKe, Beijing, China) according to manufacturer’s protocol. The successful mutations were identified by sequencing technology. The primer details are given to Supplementary Table S3.

### 2.9. Luciferase Reporter Assays

After a transfection period of 24 h, the cells and lysates were collected. A Dual-Luciferase Reporter Assay System (Promega) was used to quantify luciferase activities following the manufacturer’s instructions. Firefly luciferase activity was normalized to Renilla luciferase activity.

### 2.10. Chromatin Immunoprecipitation

In total, 1 × 10^7^ porcine GCs were collected and 1% formaldehyde was added to crosslink the protein and the chromatin. The collected cells were placed on ice and sonicated for 5 min (10 s interval on and off) by 30% output control with a 3-mm microtip. The sonicated protein-chromatin complex (~800 μL) was centrifuged at 10,000 rpm and 100 μL of the supernatant were collected as input control. Then, 400 μL of the supernatant were diluted 2.5-fold and incubated with 100 μL protein A/G-agarose (Santa Cruz, #sc-2003) by shaking for an hour at 4 °C. This mixture was then centrifuged and the purified supernatant was transferred into a new dolphin tube. Then, 10 μL (~4 μg) of anti-SMAD4 antibody or rabbit IgG antibody were added into each sample and incubated by shaking at 4 °C overnight. The reversal of crosslinking was done using 16 μL NaCl (5M) at 100°C for 10 min and the proteins were dissolved with 10 μL proteinase K at 50 °C for an hour. Then, DNA was released and precipitated with chloroform-isoamylalcohol method. Following, the enrichment of miR-1306 promoter was determined by using qRT-PCR. The primers for chromatin immunoprecipitation (ChIP) are listed in Supplementary Table S4.

### 2.11. Statistical Analysis

All results are presented as means ± SEM. Statistical analysis was carried out by using Prism 5 software (GraphPad Software). The significance of the comparison between the two groups were assessed by Two-tailed Student’s *t*-test. *p* < 0.05 was considered as significant statistical difference.

## 3. Results

### 3.1. Identification and Characterization of the 3′-UTR of the Porcine TGFBR2 Gene

To characterize the 3′ regulatory region of the porcine *TGFBR2* gene, we isolated its 3′-UTR sequence by using a RACE assay and found only one clear band, approximately 3400 bp in length (Supplementary Figure S1). Clone sequencing and sequence alignment showed that the full length 3′-UTR of the porcine *TGFBR2* gene was 2381 bp, with 94.73% nucleotide identity with the pig genome sequence (GenBank ID: XM_021071493.1), excluding 24 bp of polyA tail (PAT) sequence (Supplementary Figure S2). Porcine *TGFBR2* 3′-UTR is highly consistent with that found in mammals, such as humans (79.79%, GenBank ID: NM_001024847.2), cattle (60.47%, GenBank ID: NM_001159566), and sheep (27.55%, GenBank ID: XM_012099307). The polyA signal (PAS) (AAUAAA) is located 28 nucleotides before the PAT sequence. In addition, this region contains several classic elements, like GU-Rich Element (GRE) (UUGUU) and AU-Rich Element (ARE) (AUUUA), and a CAA Repeat ((CAA)_6_) (Supplementary Figure S2). Moreover, the miRNA response elements (MREs) for 160 candidate miRNAs were identified on the 3′-UTR of porcine *TGFBR2* mRNA (Supplementary Table S5) using an online program, RNAhybrid. Among them, miR-1306 not only had the highest binding capacity with the porcine *TGFBR2* 3′-UTR but was also observed to target porcine *TGFBR2* gene [9]. Therefore, we chose miR-1306 for further studies.

### 3.2. miR-1306 Targets the TGFBR2 3′-UTR in GCs

**Figure 1:**
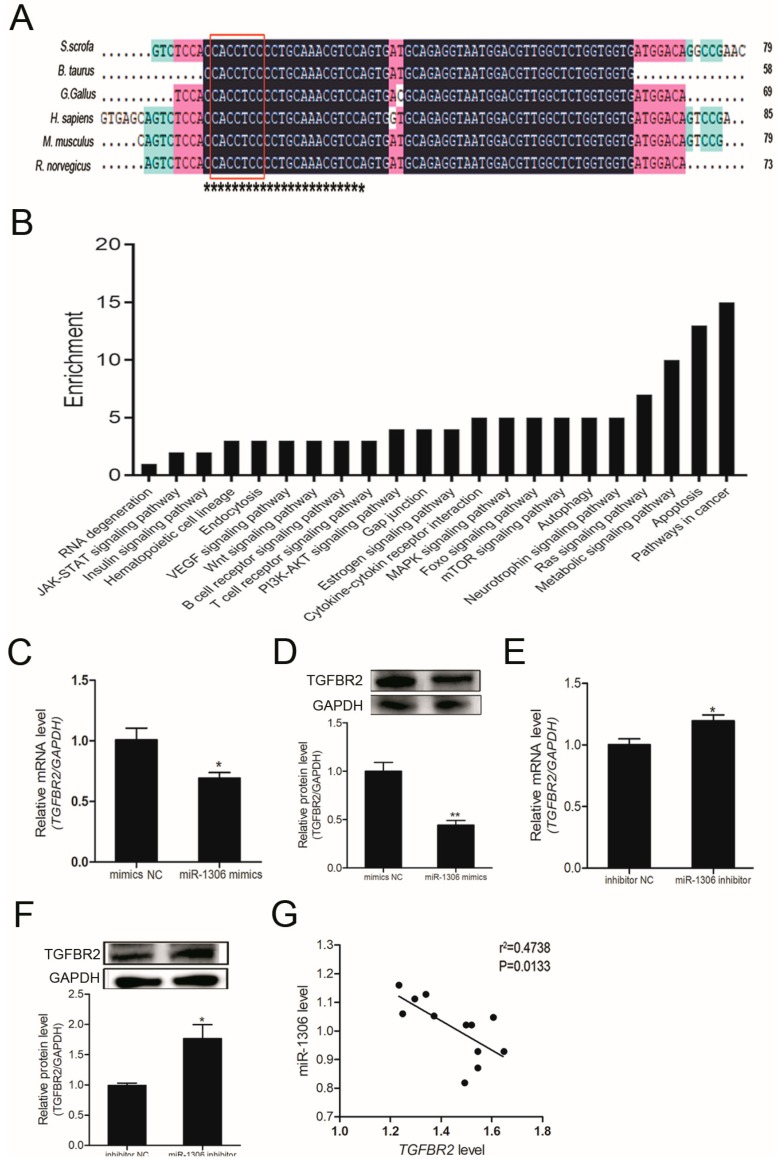
MicroRNA-1306 (miR-1306) inhibits endogenous transforming growth factor-β receptor II (TGFBR2) expression in porcine granulosa cells (GCs). (**A**) Multiple-sequence alignment of pre-miR-1306 from six different species. miR-1306 mature sequences are indicated by black asterisks. Seed sequences of miR-1306 are indicated in red boxes. S. scrofa, Sus scrofa; B. Taurus, Bos taurus; G gorilla, Gorilla gorilla; H.sapiens, Homo sapiens; M.musculus, Mus musculus; R norvegicus, Rattus norvegicus. (**B**) Kyoto Encyclopedia of Genes and Genomes (KEGG) analysis showing the pathways targeted by miR-1306. (**C**,**E**) Results of Quantitative RT-PCR (qRT-PCR) showing TGFBR2 mRNA levels and (**D**,**F**) Western blotting results showing the TGFBR2 protein levels after miR-1306 mimics or inhibitor were transfected into porcine GCs, respectively (**G**) Correlation analysis between miR-1306 and TGFBR2 (*n* = 12). The data is represented by mean ± SEM. * indicates *p* < 0.05; ** indicates *p* < 0.01.

Porcine miR-1306 or ssc-miR-1306 is positioned on the first exon of *DGCR8* gene on the chromosome 14 in pigs, similar to other vertebrates (Supplementary Figure S3). Sequence alignment revealed that pre-miR-1306 sequence was highly consistent with other vertebrates, and the mature as well as seed sequences were identical in vertebrates (Figure 1A). To understand the biological functions of miR-1306, we utilized various algorithms to predict its potential target genes. A total of 150 common target genes were found (Supplementary Table S6), which are mainly expressed during biological processes such as cancer, apoptosis, and metabolism, according to the Kyoto Encyclopedia of Genes and Genomes (KEGG) analysis (Figure 1B).

Our previous study demonstrated that the *TGFBR2* gene is directly targeted by miR-1306 in porcine GCs [9]. To evaluate whether *TGFBR2* is a functional target of miR-1306, we overexpressed and silenced the endogenous miR-1306 level in porcine GCs cultured in vitro by treating with both miR-1306 mimics and miR-1306 inhibitors (Supplementary Figure S4). qRT-PCR and Western blotting results proved that overexpression of miR-1306 significantly reduced the TGFBR2 mRNA (Figure 1C) and protein levels in GCs (Figure 1D). On the other hand, when miR-1306 was silenced in porcine GCs, the opposite result was seen (Figure 1E,F). In addition, the miR-1306 level was found to be negatively correlated with TGFBR2 mRNA level in the follicles of porcine ovaries (Figure 1G). These results suggest that miR-1306 targets the *TGFBR2* 3′-UTR in porcine GCs.

### 3.3. miR-1306 Suppresses TGF-β/SMAD Signaling Pathway by Inhibiting TGFBR2

**Figure 2:**
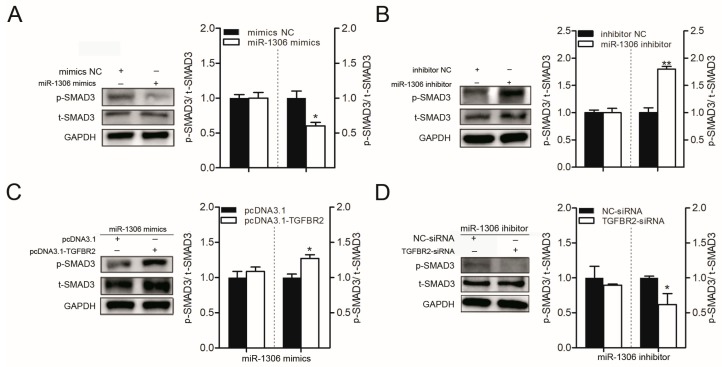
miR-1306 inactivates the transforming growth factor-β (TGF-β)/ SMA- and MAD-related protein (SMAD) signaling pathway in GCs by impairing TGFBR2. (**A**,**B**) Western blotting results showing levels of phospho-SMAD3 (p-SMAD3) and total-SMAD3 (t-SMAD3) protein after transfection of miR-1306 mimics or miR-1306 inhibitors into porcine GCs, respectively. (**C**,**D**) Western blotting results showing levels of p-SMAD3 and T-SMAD3 protein when both miR-1306 and TGFBR2 were overexpressed or silenced in porcine GCs. Each experiment has three independent repetition and results are shown as mean ± SEM. * indicates *p* < 0.05; ** indicates *p* < 0.01.

Further, we detected the levels of a downstream member of the classical TGF-β/SMAD pathway, p-SMAD3, which also acts as a marker for the pathway in porcine GCs. miR-1306 mimics or miR-1306 inhibitor were transfected into GCs and the levels of p-SMAD3 in the transfected cells were analyzed. Western blot analysis showed that p-SMAD3 levels were dramatically reduced in GCs when miR-1306 was overexpressed (Figure 2A), while p-SMAD3 levels in the GCs increased when miR-1306 was inhibited (Figure 2B). Next, we analyzed whether *TGFBR2* mediated the modulation of the miR-1306 levels during the TGF-β/SMAD signaling pathway. As expected, we discovered that *TGFBR2* overexpression rescued the miR-1306 levels by suppressing down-regulation of p-SMAD3 level induced by the miR-1306 mimics (Figure 2C), while knockdown of *TGFBR2* inhibited the miR-1306 inhibitor-mediated enhancement in p-SMAD3 level in GCs (Figure 2D). These findings suggest that miR-1306 suppresses *TGFBR2* to modulate the TGF-β/SMAD signaling pathway in porcine GCs.

### 3.4. miR-1306 Promotes GC Apoptosis by Targeting TGFBR2

**Figure 3:**
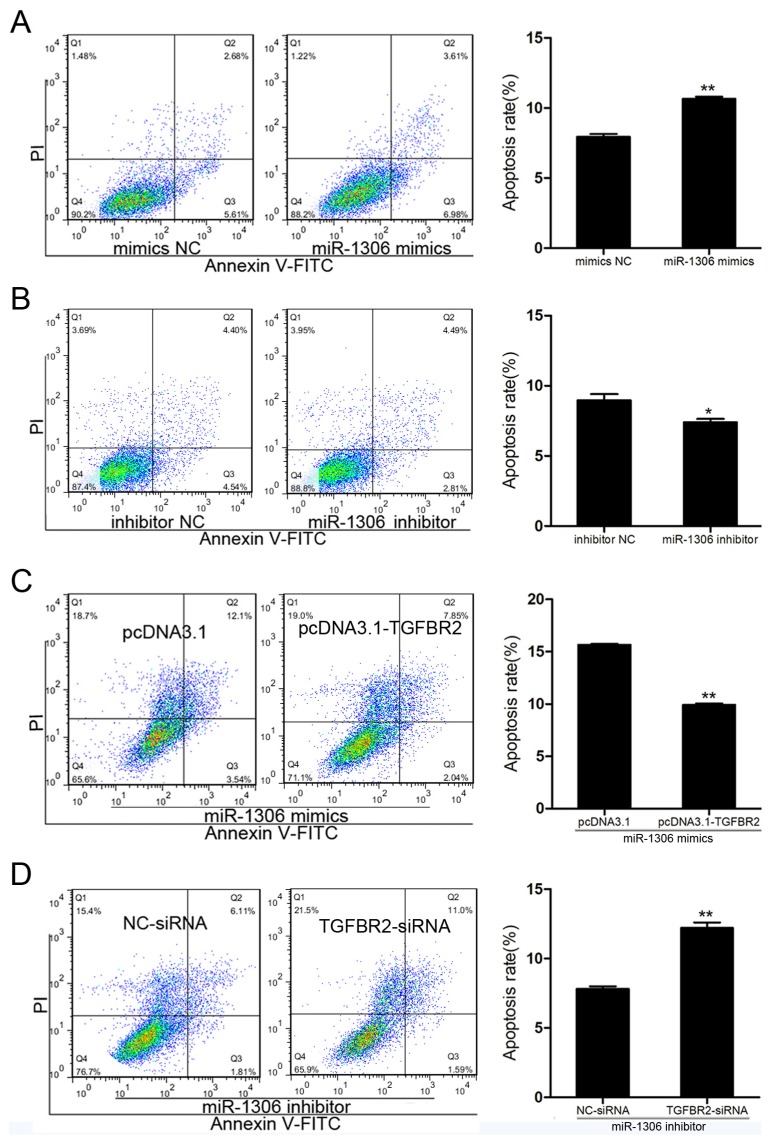
miR-1306 acts as an apoptotic factor in porcine GC through targeting TGFBR2. (**A**,**B**) Results showing the levels of GC apoptosis was (**A**) increased when cells were treated with mimic and (**B**) inhibited when cells were treated with miR-1306 inhibitor. The apoptotic cells were measured by fluorescence-activated cell sorting (FACS) (left panel) and the positive rates were calculated (right panels). (**C**,**D**) miR-1306 regulates GC apoptosis by inhibiting TGFBR2. (**C**) miR-1306 and TGFBR2 were both overexpressed or (**D**) silenced in porcine GCs. The apoptotic cells were identified by FACS (left panels) and the positive rates were calculated (right panel). Each experiment has three independent repetition and results are shown as mean ± SEM. * indicates *p* < 0.05; ** indicates *p* < 0.01.

Based on the KEGG analysis results revealing that miR-1306 may participate in apoptosis, we further investigated its role in apoptosis in porcine GCs. FACS assay showed that overexpression of miR-1306 significantly improved the GC apoptosis rate (Figure 3A), while a decrease in miR-1306 levels reduced it (Figure 3B). This suggests that miR-1306 can promote apoptosis of porcine GCs. Further, studies have shown that GC apoptosis is associated with follicular atresia. Therefore, miR-1306 level in follicles during follicular atresia of porcine ovary was measured. We discovered that the expression level of miR-1306 was remarkably increased during follicular atresia (Supplementary Figure S5). Our data shows that GC apoptosis and follicular atresia were regulated by miR-1306 in porcine ovary, both in vitro and in vivo.

Both miR-1306 mimics and *TGFBR2*-siRNA were co-transfected into porcine GCs to determine whether *TGFBR2* mediates miR-1306-induced GC apoptosis or not. Our findings suggest that silencing of *TGFBR2* could improve GC apoptosis caused by miR-1306 (Figure 3C). Furthermore, we co-transfected the GCs with miR-1306 inhibitor and pcDNA3.1-TGFBR2 and found that overexpression of *TGFBR2* decreased the GC apoptosis rate (Figure 3D). Our findings show that GC apoptosis was regulated by miR-1306 in pigs by targeting *TGFBR2*.

### 3.5. Transcription Factor SMAD4 Regulates miR-1306 Transcriptional Activity, but Does Not Depend on its Host Gene DGCR8

**Figure 4:**
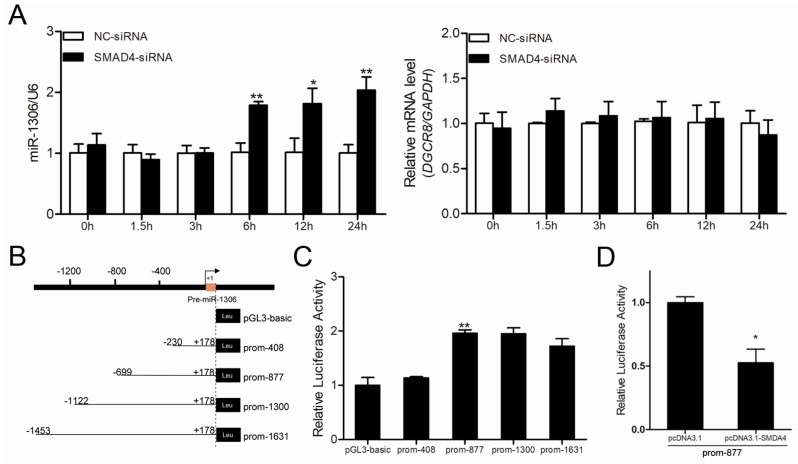
SMAD4 suppresses *miR-1306* expression by inhibiting its transcriptional activity independent of its host gene DGCR8. (**A**) The expression levels of miR-1306 (left) and its host gene DGCR8 (right) after SMAD4 silencing in porcine GCs were measured by qRT-PCR at different time intervals (0, 1.5, 3, 6, 12, 24 h). (**B**) Diagram depicting the 5′-flanking region of pre-miR-1306 and luciferase reporter vectors containing the candidate promoters of porcine miR-1306. (**C**) The recombinant vectors shown in panel **B** were transfected into porcine GCs and luciferase activities were detected. (**D**) miR-1306 promoter transcriptional activity in GCs treated with pcDNA™3.1 (pcDNA3.1)-SMAD4 was determined by Dual-Luciferase Activity Assay. Each experiment has three independent repetitions and results are shown as mean ± SEM. * indicates *p* < 0.05 and ** indicates *p* < 0.01.

To understand the mechanism by which miR-1306 was up-regulated during follicular atresia, we focused on its transcriptional regulation and promoter region. miR-1306 is transcribed from exon 1 of the porcine *DGCR8* gene and suppressed by the transcription factor SMAD4 in porcine GCs [9]. Therefore, we first investigated whether miR-1306 shares a common promoter with its host gene *DGCR8*. We found that inhibition of *SMAD4* significantly raised miR-1306 expression but had no effect on *DGCR8* expression in GCs (Figure 4A), indicating that miR-1306 has its own promoter and does not share a common promoter with its host gene *DGCR8* in porcine GCs.

Further, we identified the core promoter of the porcine miR-1306. The putative promoter of the porcine miR-1306 was predicted by using University of California, Santa Cruz (UCSC) database (Supplementary Figure S6A,B). Luciferase assay indicated that the region from −230 nt to 699 nt is the core promoter of porcine *miR-1306* gene (Figure 4B,C). Furthermore, we observed that the activity of *miR-1306* gene core promoter was dramatically suppressed by *SMAD4* overexpression (Figure 4D), suggesting that *miR-1306* gene transcriptional activity is regulated by *SMAD4*. Taken together with the results of our previous study [9], we conclude that the transcription factor SMAD4 attenuates *miR-1306* expression in porcine GCs by inhibiting its transcriptional activity.

### 3.6. SMAD4 Binds Directly to the miR-1306 Promoter to Inhibit its Transcriptional Activity

**Figure 5:**
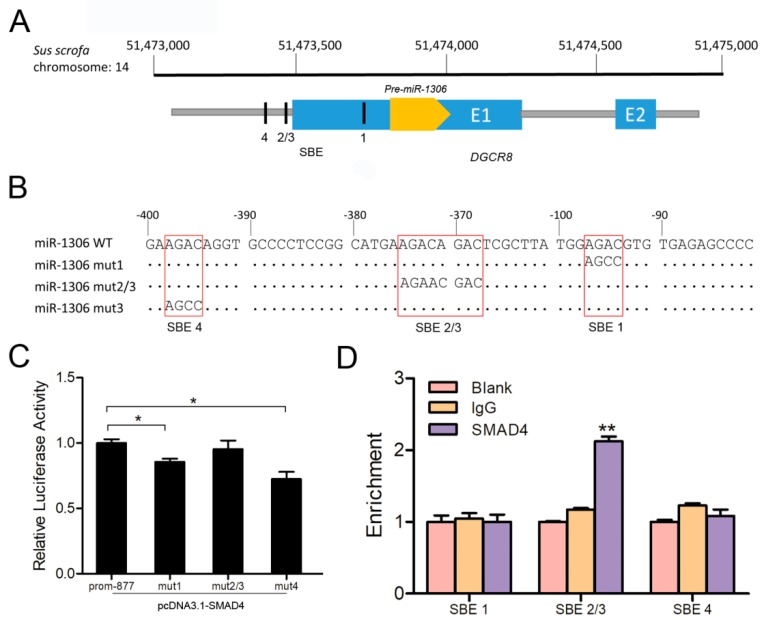
SMAD4 acts as a transcription factor and directly binds to the miR-1306 promoter. (**A**) Schematic diagram depicting the genome locations of miR-1306 (yellow) and its host gene DGCR8 (blue), and the four potential SMAD4-binding sites are shown inside the black rectangles. (**B**) The luciferase reporter vectors containing porcine miR-1306 promoter with wild-type (prom-877) or mutant-type SBE motifs, shown in the red boxes. (**C**) pcDNA3.1-SMAD4 was co-transfected with recombinant vectors in (**B**) into GCs and luciferase activity assay was performed. (**D**) Chromatin immunoprecipitation (ChIP) assays and the enrichment were determined by qRT-PCR. Each experiment was independently repeated thrice and results are presented as mean ± SEM. * *p* means <0.05 and ** means *p* < 0.01.

We also identified and characterized the core promoter of the porcine *miR-1306* gene as well as the binding regions for transcription factors such as breast cancer 1 (BRCA1), Sp1 transcription factor (SP1), and upstream transcription factor 2 (USF2) (Supplementary Figure S7). We detected four SMAD4-binding elements (SBEs) (Figure 5A), indicating that SMAD4 may act as a transcription factor and regulate *miR-1306* transcription. To confirm this, recombinant reporter vectors containing SBEs (wild-type or mutant-type) were constructed (Figure 5B) and co-transfected along with SMAD4 overexpression into the porcine GCs. Results showed that the luciferase activity of plasmids with the wild-type SBE, SBE1-mut, and SBE4-mut were dramatically enhanced after SMAD4 overexpression, but that of the SBE2/3-mut construct remains the same (Figure 5C), suggesting that SMAD4 inhibits miR-1306 transcriptional activity via SBE2/3 motif of the core promoter. Subsequently, using a ChIP assay, we confirmed that only the SBE2/3 motif within the miR-1306 promoter could specifically bind to SMAD4 (Figure 5D). Collectively, these results provide compelling evidence that SMAD4 directly interacts with SBE2/3 motif and inhibits the *miR-1306* gene transcription in GCs.

### 3.7. SMAD4 Feedback Activates TGFBR2 by Inhibiting miR-1306

**Figure 6:**
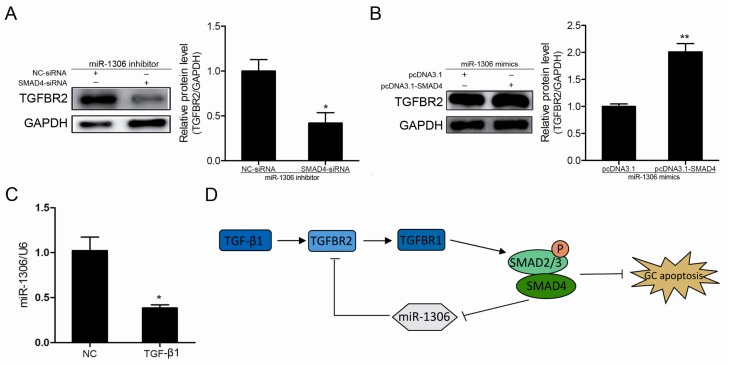
miR-1306 mediates SMAD4 feedback regulation of the classical TGF-β/SMAD signaling pathway. Results of Western blot analysis showing the TGFBR2 protein level after (**A**) SMAD4- small interfering RNAs (SMAD4-siRNA) was transfected into miR-1306-inhibited GCs and after (**B**) pcDNA3.1-SMAD4 was transfected into miR-1306-overexpressed GCs. (**C**) miR-1306 expression levels in GCs were measured after TGF-β1 (20 ng/mL) treatment. (**D**) The SMAD4/miR-1306/TGFBR2 axis is involved in SMAD4-mediated positive feedback regulation in classical TGF-β/SMAD signaling pathway. Each experiment was independently repeated thrice and the results are presented as mean ± SEM. * *p* means <0.05 and ** means *p* < 0.01.

The above results prove that *TGFBR2* is a functional target of miR-1306 and SMAD4 directly inhibits miR-1306 transcription. Therefore, miR-1306 expression may be mediated through SMAD4 feedback regulation of TGFBR2. To further confirm this, we co-transfected miR-1306 inhibitor with SMAD4-siRNA and miR-1306 mimics with pcDNA3.1-SMAD4 into the GCs. After detection, we found that the knockdown of SMAD4 dramatically suppressed miR-1306 inhibitor-induced increase in TGFBR2 protein expression (Figure 6A). In contrast, the overexpression of SMAD4 rescued the miR-1306-induced decrease in TGFBR2 protein expression (Figure 6B), indicating that SMAD4 feedback activates TGFBR2 by decreasing miR-1306. Further, we tested whether miR-1306 expression levels are affected by the TGF-β/SMAD signaling pathway. Our results showed that the miR-1306 levels sharply reduced in the GCs treated with 20 ng/mL of TGF-β1 (TGF-β/SMAD signaling pathway activation) (Figure 6C). Therefore, miR-1306 can interact with SMAD4 and TGFBR2, which further participates in SMAD4-mediated positive feedback regulation of the classical TGF-β/SMAD signaling pathway in GCs.

## 4. Discussion

TGFBR2 is known to be the first receptor to be activated in the classical TGF-β/SMAD signaling pathway and it mediates the effects of TGF-β ligands (TGF-β1) by forming a receptor–receptor complex with TGFBR1, also named ALK5. Actually, ALK1 could also act as partner of TGFBR2 [25] but ALK1 mediates the phosphorylation of SMAD1/5/8 [26] and activates SMAD2/3 only in the presence of ALK5, which is essential for SMAD2/3 phosphorylation [27]. In this study, we identified and characterized the 3’-UTR of porcine *TGFBR2* gene and observed several response elements such as ARE, GREs, and MREs for the first time. It is well known that the levels of mRNAs are largely controlled by mRNA decay mechanisms including mRNA decay induced or inhibited proteins and small regulatory RNAs [28,29]. Furthermore, some *cis*-elements in the mRNAs 3’-UTRs are also known to mediate the regulation of mRNA decay, such as PAT [29,30], ARE [31], iron-responsive element (IRE) [32], GRE [33], and MRE [34]. Many studies have demonstrated that the expression of *TGFBR2* is modulated by MREs and their miRNAs in various cell types, such as miR-145 in vascular smooth muscle cells [35], miR-9-5p in hepatic stellate cells [36], miR-9 and miR-9-5p fibroblasts (HCFs) [6,37], miR-520e, miR-9-5p, and miR-135b in cancer cells [38,39,40]. Notably, miR-143 [23], miR-130a, miR-425, and miR-1306 [9] are known to target *TGFBR2* in porcine GCs. However, currently there are no studies investigating the involvement of other cis-elements like PAT, ARE, and GRE in *TGFBR2* mRNA decay.

Our previous study proved that miR-1306 targets the *TGFBR2* 3′UTR in pigs [9]. In this study, we further confirmed that *TGFBR2* is a functional target of miR-1306 in porcine GCs and miR-1306 promotes GC apoptosis by impairing *TGFBR2* and inactivating TGFBR2-dependent TGF-β/SMAD signaling pathway. *TGFBR2* and TGFBR2-dependent TGF-β/SMAD pathway participate in GC apoptosis, as observed in several studies conducted on mammals. In pigs, for instance, TGFBR2 is a crucial repressor of GC apoptosis [9]. Furthermore, TGF-β1 could activate TGF-β/SMAD pathway and inhibit GC apoptosis, while blocking the TGF-β/SMAD signaling pathway by a specific inhibitor such as LY2157299, which can promote GC apoptosis [23,41]. Similarly, the TGF-β/SMAD signaling pathway is also known to play an important role in GC apoptosis in mammals such as humans and rodents [42,43,44,45]. While the function of miRNA-1306 has been seldom reported, studies have strongly suggested that miRNA-1306 can target and inhibit ADAM10 gene, a key gene of Alzheimer’s disease (AD) [46]. Our findings define the function of miR-1306 in GC apoptosis as well as follicular atresia in mammals. The results of our study also proved that miR-1306 might serve as a molecular stimulator for female fertility. Moreover, our findings reveal the mechanism of action of miR-1306 in GCs as well as its ovarian functions.

mRNAs are ubiquitous in the genome of eukaryotes and occur within intragenic regions such as introns and exons, or within intergenic regions. The proportion of intragenic miRNAs varies from approximately 33% in pigs to 55% in mouse, and most of them are intronic miRNAs, while very few are exonic miRNAs [47]. The transcriptional regulation and functions of intragenic miRNAs and their host genes has been a hot topic of research [48,49]. Most intronic miRNAs are seen to co-express and are functionally consistent with their host genes [48,50,51]. A recent study showed that the transcription of intronic miRNAs does not depend on host genes and their functionally coordination is less extensive than expected [52]. However, the relationship between exonic miRNAs and their host genes still remains unknown [53]. Our study suggests that although the transcription of miR-1306, which is an exonic miRNA, is suppressed by the transcription factor SMAD4, its host gene *DGCR8* is not regulated by SMAD4 in porcine GCs. Furthermore, we also demonstrated that miR-1306 has its own internal promoter that is independent of the host gene, and that SMAD4 serves as a negative regulatory factor and suppresses miR-1306 by directly binding to SBE motif within this promoter region. Therefore, our findings provide strong evidence that the exonic miR-1306 is transcribed independently of the host gene in porcine GCs. This not only clarifies the relationship between miR-1306 and its host gene *DGCR8* but also lays a foundation for the further understanding of its regulatory mechanism.

Notably, our study shows that *miR-1306* is not only regulated by SMAD4 but also targets *TGFBR2*, a key receptor upstream of SMAD4, suggesting that miR-1306 mediates SMAD4-positive feedback regulation of the classical TGF-β/SMAD signaling pathway which was discovered in 2016 [54]. Recently, a few reports have revealed the mechanism of this feedback regulation, which was found to be mediated by the miR-122-5p–TGFBR2 axis in mouse skeletal muscle fibrosis [55] or by the miR-425-TGFBR2 axis in porcine GCs [9]. Among the classical TGF-β/SMAD signaling pathway, SMAD2 and SMAD3, another two SMAD proteins, were found to be feedback regulators in as early as 2001 and 1999, respectively [56,57]. This feedback regulation has recently been demonstrated to be achieved through the miRNA–TGFBR2 axis (e.g., the miRNA-520e–TGFBR2 axis) [38]. This suggested that *TGFBR2* is an important target in the feedback regulation of the SMAD protein from the classical TGF-β/SMAD signaling pathway. Another key receptor, *TGFBR1*, is a primary target of SMAD7 (an inhibitory SMAD protein [58]), which further regulated the classical TGF-β/SMAD signaling pathway through negative feedback regulation. SMAD7 has been seen to regulate *TGFBR1* through negative feedback in many ways [59]. In one of our recent studies, we have shown that SMAD7 directly binds to the promoter of *TGFBR1* and further inhibits its transcription in porcine GCs [60].

## 5. Conclusions

In conclusion, we proved that the pro-apoptotic factor miR-1306 controls porcine GC apoptosis by targeting *TGFBR2* and inactivating the TGF-β/SMAD signaling pathway. Our findings pave the way for a potential non-hormonal target or drug therapy candidate for improving female fertility. Moreover, the transcription factor SMAD4 was identified as a transcriptional regulator of the exonic miR-1306, but this process is not independent of its host gene *DGCR8*. Our findings also reveal a feedback mechanism of the classical TGF-β/SMAD signaling pathway (Figure 6D) and provide novel insights into feedback regulation within the classical TGF-β/SMAD signaling pathway.

## Supplementary Materials

The following are available online at https://www.mdpi.com/2073-4409/8/4/298/s1, Supplementary Figure S1: Rapid amplification of cDNA ends (RACE) of TGFBR2 3’-UTR, Supplementary Figure S2: Analysis for the TGFBR2 3′-UTR, Supplementary Figure S3: The Positioning analysis of miR-1306 from five different species, Supplementary Figure S4: Expression levels of miR-1306 in p GCs after miR-1306 mimics or inhibitor transfection, Supplementary Figure S5: The expression of miR-1306 during follicluar atresia of porcine ovary, Supplementary Figure S6: Identification of the putative promoter of miR-1306 in pigs, Supplementary Figure S7: Identification of SBE motifs in the porcine miR-1306 gene promoter, Supplementary Table S1: The miRNA response elements (MREs) in the 3’UTR of porcine TGFBR2 gene, Supplementary Table S2: The potential target genes of miR-1306, Supplementary Table S3: Oligonucleotide sequences used in this study, Supplementary Table S4: Primers designed for reverse-transcription and QRT-PCR, Supplementary Table S5: Primers used for plasmids construction and mutation, Supplementary Table S6: Primers for chromatin immunoprecipitation.

## References

[1] Andl T., Le Bras G.F., Richards N.F., Allison G.L., Loomans H.A., Washington M.K., Andl C.D. (2014). Concerted loss of TGFβ-mediated proliferation control and E-cadherin disrupts epithelial homeostasis and causes oral squamous cell carcinoma. Carcinogenesis.

[2] Mullen A.C., Wrana J.L. (2017). TGF-beta Family Signaling in Embryonic and Somatic Stem-Cell Renewal and Differentiation. Cold Spring Harb. Perspect. Biol..

[3] Massague J., Gomis R.R. (2006). The logic of TGFbeta signaling. FEBS Lett..

[4] Heldin C.H., Landstrom M., Moustakas A. (2009). Mechanism of TGF-beta signaling to growth arrest, apoptosis, and epithelial-mesenchymal transition. Curr. Opin. Cell Biol..

[5] Hill C.S. (2016). Transcriptional control by the SMADs. Cold Spring Harb. Perspect. Biol..

[6] Ahn Y.H., Kim T.H., Choi H., Bae C.H., Yang Y.M., Baek J.A., Cho E.S. (2015). Disruption of Tgfbr2 in odontoblasts leads to aberrant pulp calcification. J. Dent. Res..

[7] Biswas S., Trobridge P., Romero-Gallo J., Billheimer D., Myeroff L.L., Willson J.K., Grady W.M. (2008). Mutational inactivation of TGFBR2 in microsatellite unstable colon cancer arises from the cooperation of genomic instability and the clonal outgrowth of transforming growth factor beta resistant cells. Genes Chromosom. Cancer.

[8] Ostapoff K.T., Euhus D., Xie X.J., Rao M., Moldrem A., Rao R. (2011). Axillary lymph node dissection for breast cancer utilizing Harmonic Focus(R). World J. Surg. Oncol..

[9] Du X., Pan Z., Li Q., Liu H., Li Q. (2018). SMAD4 feedback regulates the canonical TGF-beta signaling pathway to control granulosa cell apoptosis. Cell Death Dis..

[10] Dhasarathy A., Phadke D., Mav D., Shah R.R., Wade P.A. (2011). The transcription factors Snail and Slug activate the transforming growth factor-beta signaling pathway in breast cancer. PLoS ONE.

[11] Bizet A.A., Tran-Khanh N., Saksena A., Liu K., Buschmann M.D., Philip A. (2012). CD109-mediated degradation of TGF-beta receptors and inhibition of TGF-beta responses involve regulation of SMAD7 and Smurf2 localization and function. J. Cell Biochem..

[12] Murai F., Koinuma D., Shinozaki-Ushiku A., Fukayama M., Miyaozono K., Ehata S. (2015). EZH2 promotes progression of small cell lung cancer by suppressing the TGF-beta-Smad-ASCL1 pathway. Cell Discov..

[13] Garcia D.A., Baek C., Estrada M.V., Tysl T., Bennett E.J., Yang J., Chang J.T. (2018). USP11 enhances TGFbeta-Induced epithelial-mesenchymal plasticity and human breast cancer metastasis. Mol. Cancer Res..

[14] Markowitz S., Wang J., Myeroff L., Parsons R., Sun L., Lutterbaugh J., Vogelstein B. (1995). Inactivation of the type II TGF-beta receptor in colon cancer cells with microsatellite instability. Science.

[15] Li J., Ao J., Li K., Zhang J., Li Y., Zhang L., Huang L. (2016). ZNF32 contributes to the induction of multidrug resistance by regulating TGF-beta receptor 2 signaling in lung adenocarcinoma. Cell Death Dis..

[16] Fan Y., Gao Y., Rao J., Zhang F., Wang K., Zhang C. (2017). YAP-1 promotes tregs differentiation in hepatocellular carcinoma by enhancing TGFBR2 transcription. Cell Physiol. Biochem..

[17] Zhou B., Guo W., Sun C., Zhang B., Zheng F. (2018). Linc00462 promotes pancreatic cancer invasiveness through the miR-665/TGFBR1-TGFBR2/SMAD2/3 pathway. Cell Death Dis..

[18] Shi Y., Yang X., Xue X., Sun D., Cai P., Song Q., Qin L. (2018). HANR promotes hepatocellular carcinoma progression via miR-214/EZH2/TGF-beta axis. Biochem. Biophys. Res. Commun..

[19] Liu J.J., Zhang X., Wu X.H. (2018). miR-93 promotes the growth and invasion of prostate cancer by upregulating its target genes TGFBR2, ITGB8, and LATS2. Mol. Ther. Oncolytics.

[20] van Kampen J.G., van Hooij O., Jansen C.F., Smit F.P., van Noort P.I., Schultz I., Verhaegh G.W. (2017). miRNA-520f reverses epithelial-to-mesenchymal transition by targeting ADAM9 and TGFBR2. Cancer Res..

[21] Yao W., Li Y., Han L., Ji X., Pan H., Liu Y., Ni C. (2018). The CDR1as/miR-7/TGFBR2 axis modulates EMT in silica-induced pulmonary fibrosis. Toxicol. Sci..

[22] Wang H., Wang G., Gao Y., Zhao C., Li X., Zhang F., Wu B. (2018). Lnc-SNHG1 activates the TGFBR2/SMAD3 and RAB11A/Wnt/beta-catenin pathway by sponging MiR-302/372/373/520 in invasive pituitary tumors. Cell Physiol. Biochem..

[23] Du X., Zhang L., Li X., Pan Z., Liu H., Li Q. (2016). TGF-beta signaling controls FSHR signaling-reduced ovarian granulosa cell apoptosis through the SMAD4/miR-143 axis. Cell Death Dis..

[24] Liu J., Du X., Zhou J., Pan Z., Liu H., Li Q. (2014). MicroRNA-26b functions as a proapoptotic factor in porcine follicular Granulosa cells by targeting Sma-and Mad-related protein 4. Biol. Reprod..

[25] Derynck R., Zhang Y.E. (2003). Smad-dependent and Smad-independent pathways in TGF-beta family signalling. Nature.

[26] Zhang H., Du L., Zhong Y., Flanders K.C., Roberts J.D. (2017). Transforming growth factor-beta stimulates Smad1/5 signaling in pulmonary artery smooth muscle cells and fibroblasts of the newborn mouse through ALK1. Am. J. Physiol. Lung Cell Mol. Physiol..

[27] Goumans M.J., Valdimarsdottir G., Itoh S., Rosendahl A., Sideras P., ten Dijke P. (2002). Balancing the activation state of the endothelium via two distinct TGF-beta type I receptors. EMBO J..

[28] McFarland A.P., Horner S.M., Jarret A., Joslyn R.C., Bindewald E., Shapiro B.A., Savan R. (2014). The favorable IFNL3 genotype escapes mRNA decay mediated by AU-rich elements and hepatitis C virus-induced microRNAs. Nat. Immunol..

[29] Tsai T.L., Lin C.H., Lin C.N., Lo C.Y., Wu H.Y. (2018). Interplay between the Poly(A) Tail, Poly(A)-Binding Protein, and Coronavirus Nucleocapsid Protein Regulates Gene Expression of Coronavirus and the Host Cell. J. Virol..

[30] Kuba K. (2018). New aspects of poly(A) tail shortening of mRNA in controlling heart functions. Nihon Yakurigaku Zasshi.

[31] Yanagawa-Matsuda A., Mikawa Y., Habiba U., Kitamura T., Yasuda M., Towfik-Alam M., Higashino F. (2018). Oncolytic potential of an E4-deficient adenovirus that can recognize the stabilization of AU-rich element containing mRNA in cancer cells. Oncol. Rep..

[32] Zhou Z.D., Tan E.K. (2017). Iron regulatory protein (IRP)-iron responsive element (IRE) signaling pathway in human neurodegenerative diseases. Mol. Neurodegener..

[33] Vlasova-St. Louis I., Bohjanen P.R., Vlasova-St. Louis I., Bohjanen P.R. (2014). Post-transcriptional regulation of cytokine signaling by AU-rich and GU-rich elements. J. Interferon Cytokine Res..

[34] Guo H., Ingolia N.T., Weissman J.S., Bartel D.P. (2010). Mammalian microRNAs predominantly act to decrease target mRNA levels. Nature.

[35] Zhao N., Koenig S.N., Trask A.J., Lin C.H., Hans C.P., Garg V., Lilly B. (2015). MicroRNA miR145 regulates TGFBR2 expression and matrix synthesis in vascular smooth muscle cells. Circ. Res..

[36] Yu F., Chen B., Fan X., Li G., Dong P., Zheng J. (2017). Epigenetically-regulated MicroRNA-9-5p suppresses the activation of hepaticd stellate cells via TGFBR1 and TGFBR2. Cell Physiol. Biochem..

[37] Li J., Dai Y., Su Z., Wei G. (2016). MicroRNA-9 inhibits high glucose-induced proliferation, differentiation and collagen accumulation of cardiac fibroblasts by down-regulation of TGFBR2. Biosci. Rep..

[38] Kucuksayan H., Akgun S., Ozes O.N., Alikanoglu A.S., Yildiz M., Dal E., Akca H. (2018). TGF-beta-SMAD-miR-520e axis regulates NSCLC metastasis through a TGFBR2-mediated negative feedback loop. Carcinogenesis.

[39] Li G., Wu F., Yang H., Deng X., Yuan Y. (2017). MiR-9-5p promotes cell growth and metastasis in non-small cell lung cancer through the repression of TGFBR2. Biomed. Pharmacother..

[40] Li J., Liang H., Bai M., Ning T., Wang C., Fan Q., Zen K. (2015). miR-135b promotes cancer progression by targeting transforming growth factor beta receptor II (TGFBR2) in colorectal cancer. PLoS ONE.

[41] Zhou J., Liu J., Pan Z., Du X., Li X., Ma B., Liu H. (2015). The let-7g microRNA promotes follicular granulosa cell apoptosis by targeting transforming growth factor-beta type 1 receptor. Mol. Cell Endocrinol..

[42] Li Y., Jin Y., Liu Y., Shen C., Dong J., Xu J. (2013). SMAD3 regulates the diverse functions of rat granulosa cells relating to the FSHR/PKA signaling pathway. Reproduction.

[43] Han X., Xue R., Yuan H.J., Wang T.Y., Lin J., Zhang J., Tan J.H. (2017). MicroRNA-21 plays a pivotal role in the oocyte-secreted factor-induced suppression of cumulus cell apoptosis. Biol. Reprod..

[44] Zhou J., Lei B., Li H., Zhu L., Wang L., Tao H., Li F. (2017). MicroRNA-144 is regulated by CP2 and decreases COX-2 expression and PGE2 production in mouse ovarian granulosa cells. Cell Death Dis..

[45] Chu Y.L., Xu Y.R., Yang W.X., Sun Y. (2018). The role of FSH and TGF-beta superfamily in follicle atresia. Aging (Albany NY).

[46] Augustin R., Endres K., Reinhardt S., Kuhn P.H., Lichtenthaler S.F., Hansen J., Trümbach D. (2012). Computational identification and experimental validation of microRNAs binding to the Alzheimer-related gene ADAM10. BMC Med. Genet..

[47] Paczynska P., Grzemski A., Szydlowski M. (2015). Distribution of miRNA genes in the pig genome. BMC Genet..

[48] Hinske L.C.G., Galante P.A., Kuo W.P., Ohno-Machado L. (2010). A potential role for intragenic miRNAs on their hosts’ interactome. BMC Genom..

[49] França G.S., Hinske L.C., Galante P.A., Vibranovski M.D. (2017). Unveiling the impact of the genomic architecture on the evolution of vertebrate microRNAs. Front. Genet..

[50] Guan H., Wei G., Wu J., Fang D., Liao Z., Xiao H., Li Y. (2013). Down-regulation of miR-218-2 and its host gene SLIT3 cooperate to promote invasion and progression of thyroid cancer. J. Clin. Endocrinol. Metab..

[51] Zhang H., Zhang L., Sun T. (2018). Cohesive regulation of neural progenitor development by microRNA miR-26, its host gene ctdsp and target gene Emx2 in the mouse embryonic cerebral cortex. Front. Mol. Neurosci..

[52] Steiman-Shimony A., Shtrikman O., Margalit H. (2018). Assessing the functional association of intronic miRNAs with their host genes. RNA.

[53] Elton T.S., Selemon H., Elton S.M., Parinandi N.L. (2013). Regulation of the MIR155 host gene in physiological and pathological processes. Gene.

[54] Elton T.S., Selemon H., Elton S.M., Parinandi N.L. (2016). A comprehensive transcriptomic view on the role of SMAD4 gene by RNAi-mediated knockdown in porcine follicular granulosa cells. Reproduction.

[55] Sun Y., Wang H., Li Y., Liu S., Chen J., Ying H. (2018). miR-24 and miR-122 Negatively regulate the transforming growth factor-beta/smad signaling pathway in skeletal muscle fibrosis. Mol. Ther. Nucleic Acids.

[56] Ito Y., Sarkar P., Mi Q., Wu N., Bringas P., Liu Y., Chai Y. (2001). Overexpression of Smad2 reveals its concerted action with Smad4 in regulating TGF-beta-mediated epidermal homeostasis. Dev. Biol..

[57] Ashcroft G.S., Yang X., Glick A.B., Weinstein M., Letterio J.J., Mizel D.E., Roberts A.B. (1999). Mice lacking Smad3 show accelerated wound healing and an impaired local inflammatory response. Nat. Cell Biol..

[58] Yan X., Xiong X., Chen Y.G. (2018). Feedback regulation of TGF-beta signaling. Acta Biochim. Biophys. Sin. (Shanghai).

[59] Miyazawa K., Miyazono K. (2017). Regulation of TGF-beta family signaling by inhibitory Smads. Cold Spring Harb. Perspect. Biol..

[60] Yao W., Pan Z., Du X., Zhang J., Li Q. (2018). miR-181b-induced SMAD7 downregulation controls granulosa cell apoptosis through TGF-beta signaling by interacting with the TGFBR1 promoter. J. Cell Physiol..

