# Supplementary material for: miR-1306 Mediates the Feedback Regulation of the TGF-β/SMAD Signaling Pathway in Granulosa Cells
Source: Cells. 2019 Mar 31;8(4):298. doi: 10.3390/cells8040298 (PMC6523565; doi:10.3390/cells8040298)

Supplementary Figure Legends

**Supplementary Figure 1.** Rapid amplification of cDNA ends (RACE) of TGFBR2 3’-UTR. Analysis products on agarose gel, the 5 Kb DNA size marker are shown in extreme left lane marked by letter M.

**Supplementary Figure 2.** Analysis for the TGFBR2 3'-UTR. The Poly (A) is indicated by a black box. GREs, ARE and CAA repeat are underlined.

**Supplementary Figure 3.** The Positioning analysis of miR-1306 from five different species.

**Supplementary Figure 4.** Expression levels of miR-1306 in porcine GCs after miR-1306 mimics or inhibitor transfection.

**Supplementary Figure 5.** The expression of miR-1306 during follicluar atresia of porcine ovary.

**Supplementary Figure 6.** Identification of the putative promoter of miR-1306 in pigs. (A) Location of miR-1306 and the host gene DGCR8 on the genome of the pig or other species deposited in NCBI database and UCSC Genome databases. (B) Potential promoters are marked by arrows.

**Supplementary Figure 7.** Identification of SBE motifs in the porcine miR-1306 gene promoter. Nucleotide numbering is relative to +1 at the beginning site of pre-miR-1306. The SBE motifs are underlined. Pre-miR-1306 is indicated by a black box.

**Supplementary Figure 1**


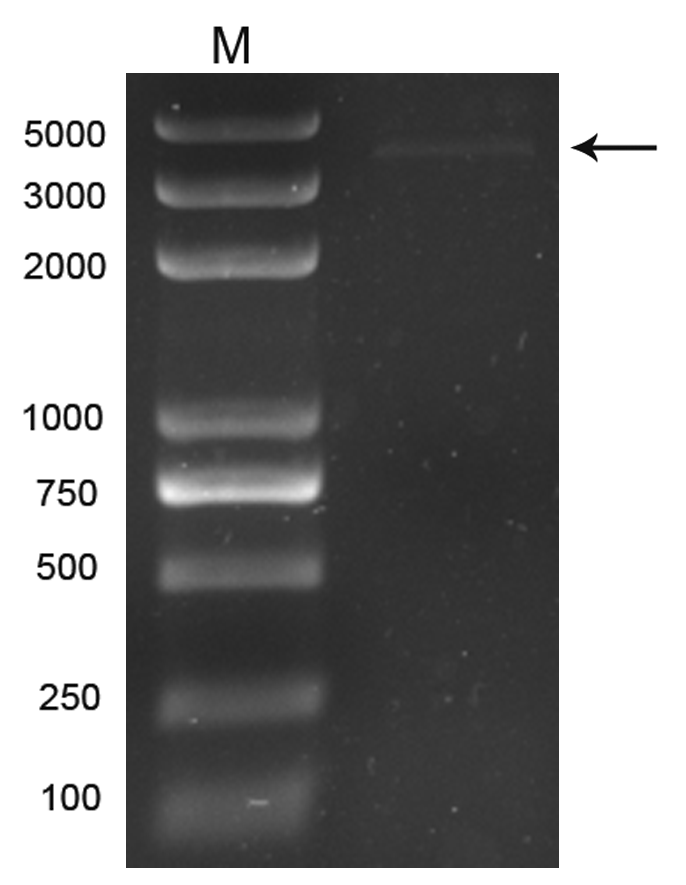


**Supplementary Figure 2**


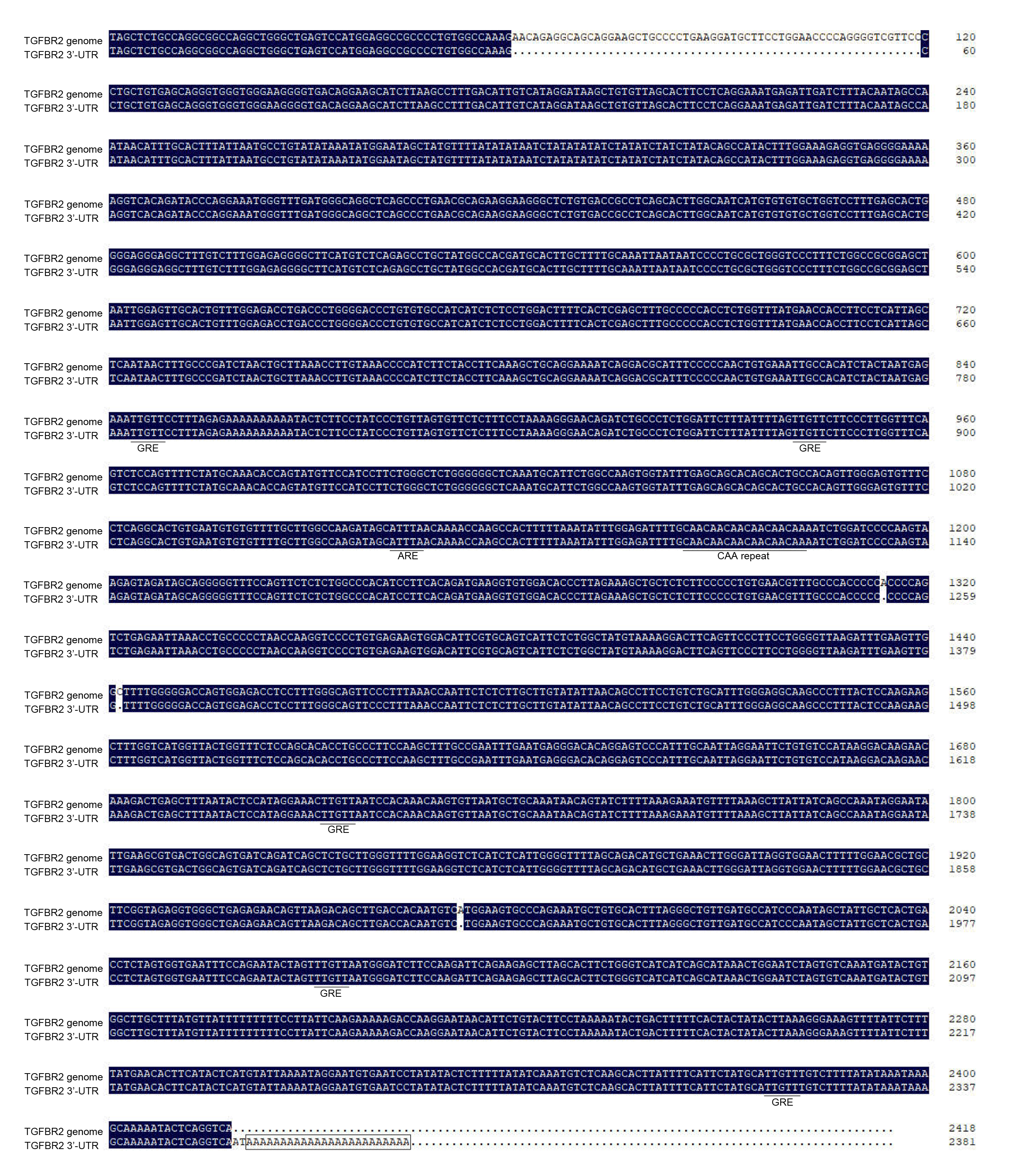


**Supplementary Figure 3**


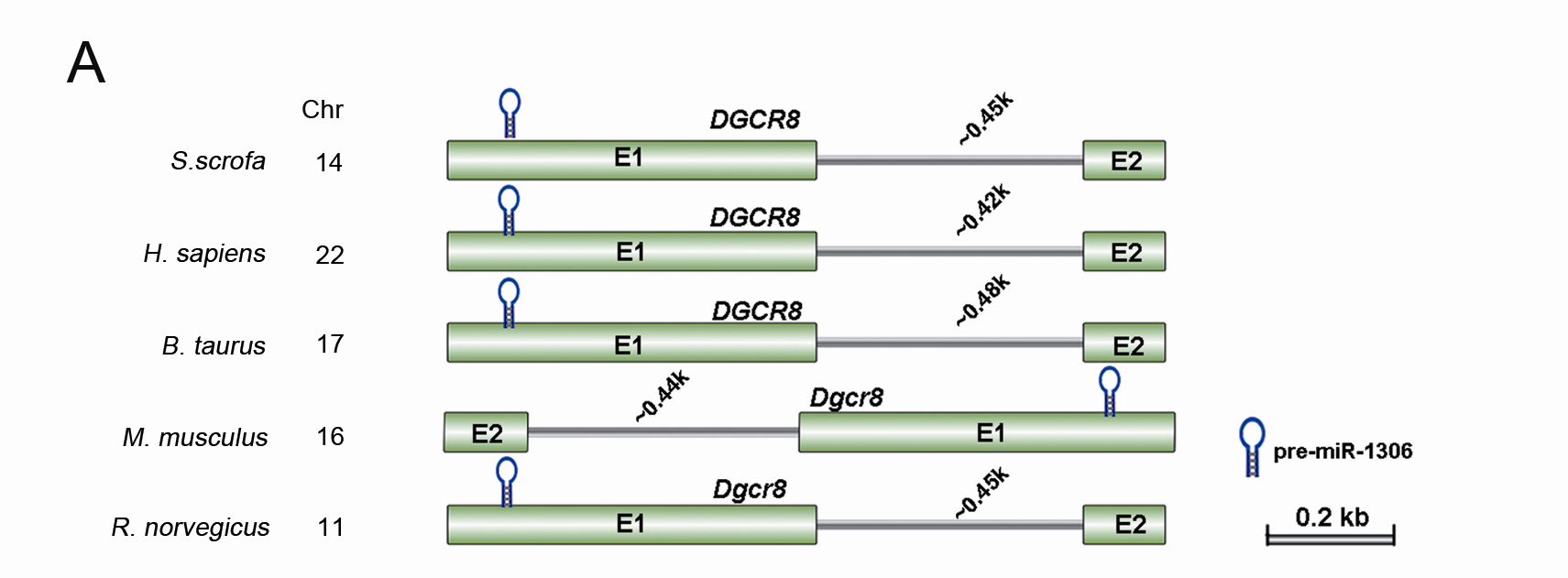


**Supplementary Figure 4**


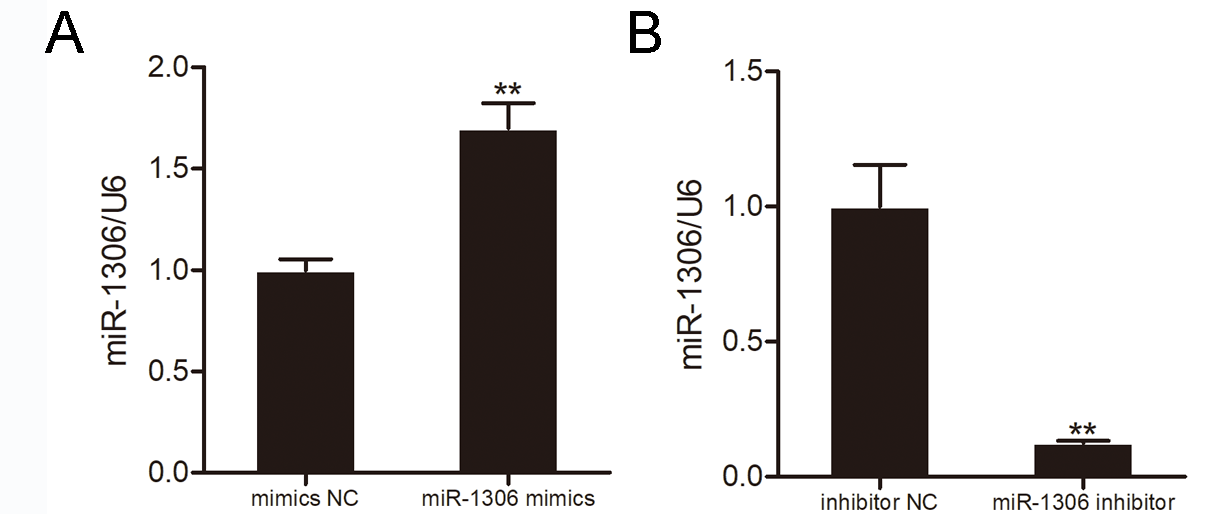


**Supplementary Figure 5**


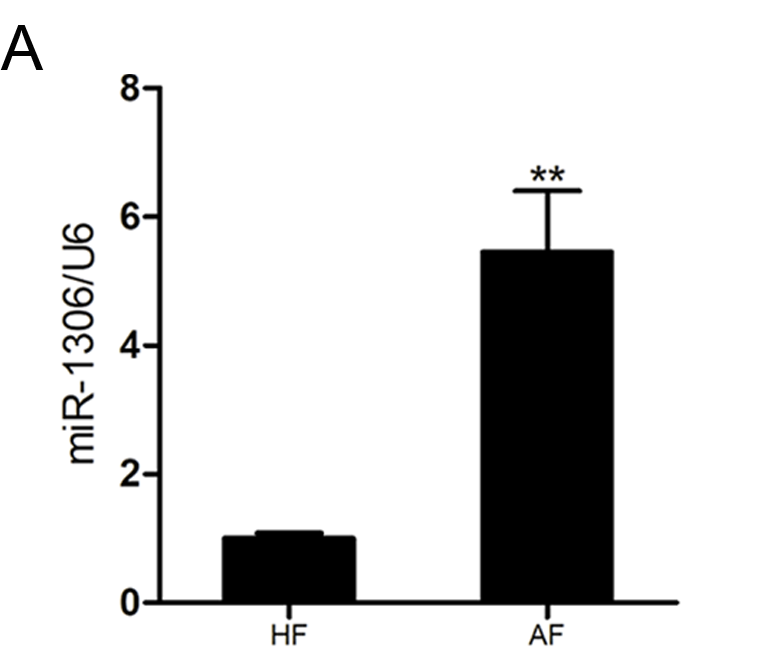


**Supplementary Figure 6**


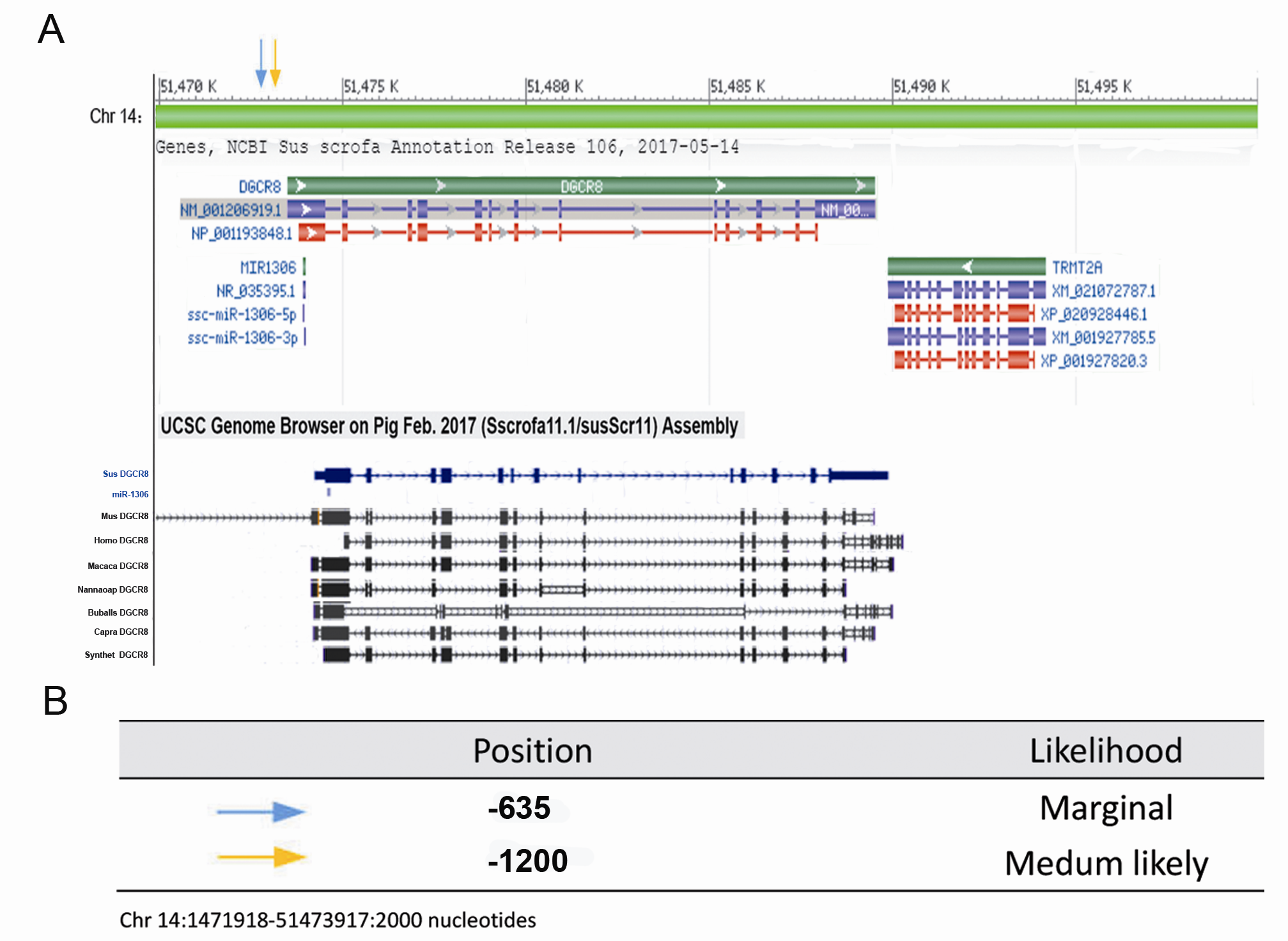


**Supplementary Figure 7**


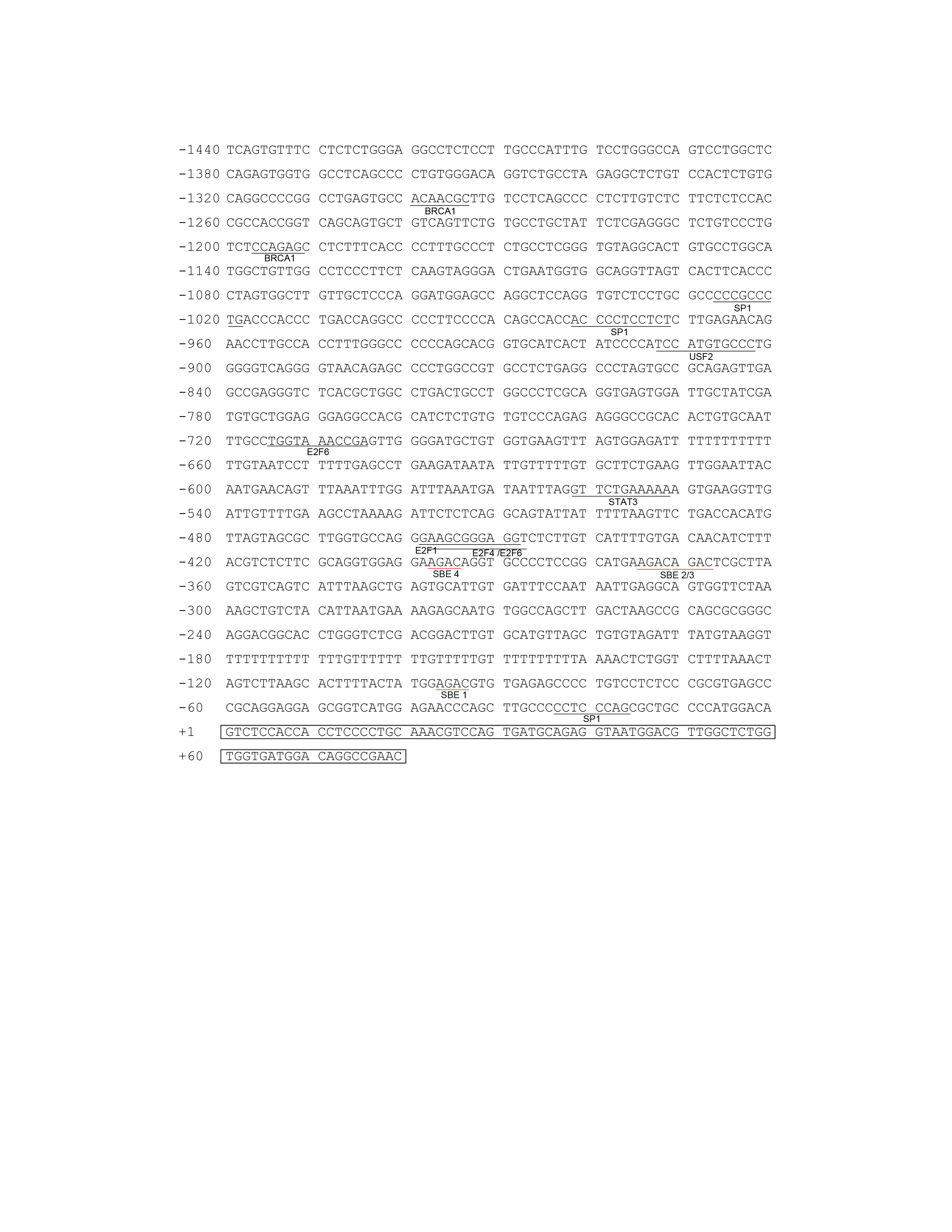

Supplement: Supplementary file 1 [file cells-08-00298-s001.zip › cells-442898-supplementary/Supplementary File/Supplementary Figure Legends.docx]
