# Supplementary material for: miR-1306 Mediates the Feedback Regulation of the TGF-β/SMAD Signaling Pathway in Granulosa Cells
Source: Cells. 2019 Mar 31;8(4):298. doi: 10.3390/cells8040298 (PMC6523565; doi:10.3390/cells8040298)
Supplement: Supplementary file 1 [file cells-08-00298-s001.zip › cells-442898-supplementary/Supplementary File/Supplementary Table legends.docx]

**Supplementary Table legends**

**Supplementary Table 1** The miRNA response elements (MREs) in the 3’UTR of porcine TGFBR2 gene.

**Supplementary Table 2** The potential target genes of miR-1306.

**Supplementary Table 3** Oligonucleotide sequences used in this study.

**Supplementary Table 4** Primers designed for reverse-transcription and QRT-PCR.

**Supplementary Table 5** Primers used for plasmids construction and mutation.

**Supplementary Table 6** Primers for chromatin immunoprecipitation.

**Supplementary Table 1**

| **miRNA** | **Location** | **MFE**  **(kcal/mol)** |
| --- | --- | --- |
| miR-1306 | 74-81 | -31.7 |
| miR-6858-5p | 501-519 | -30.6 |
| miR-6794-3p | 1746-1779 | -28.9 |
| miR-30b-3p | 2364-2403 | -28.8 |
| miR-504-3p | 499-590 | -28.3 |
| miR-3135b | 468-491 | -28.2 |
| miR-3670 | 1754-1791 | -28 |
| miR-6824-5p | 501-519 | -27.8 |
| miR-3132 | 925-964 | -27.7 |
| miR-4648 | 1566-1628 | -27.7 |
| miR-6769b-5p | 511-538 | -27.6 |
| miR-425 | 344-349 | -27.5 |
| miR-6782-3p | 1564-1597 | -27.2 |
| miR-34a-5p | 455-479 | -26.1 |
| miR-6076 | 512-532 | -26 |
| miR-4329 | 1387-1423 | -25.8 |
| miR-6731-5p | 469-492 | -25.8 |
| miR-6829-5p | 1378-1431 | -25.7 |
| miR-449b-5p | 458-487 | -25.6 |
| miR-4717-3p | 447-469 | -25.5 |
| miR-365a-5p | 1272-1295 | -25.4 |
| miR-3191-3p | 449-475 | -25.2 |
| miR-550a-5p | 1017-1024 | -25 |
| miR-143 | 1931-1936 | -24.8 |
| miR-6837-5p | 511-533 | -24.6 |
| miR-6820-5p | 1271-1401 | -24.5 |
| miR-877-5p | 502-519 | -24.4 |
| miR-550b-2-5p | 1017-1024 | -24.3 |
| miR-31-5p | 127-147 | -24.1 |
| miR-422a | 506-546 | -24.1 |
| miR-4474-3p | 451-470 | -24.1 |
| miR-449a | 458-486 | -24.0 |
| miR-619-5p | 447-465 | -23.9 |
| miR-657 | 1558-1606 | -23.9 |
| miR-378c | 514-546 | -23.8 |
| miR-4428 | 2083-2100 | -23.8 |
| miR-130a | 1782-1788 | -23.7 |
| miR-4260 | 447-457 | -23.7 |
| miR-198 | 506-529 | -23.6 |
| miR-4432 | 2087-2106 | -23.6 |
| miR-1182 | 1276-129 | -23.5 |
| miR-4487 | 449-466 | -23.5 |
| miR-6849-5p | 1273-1313 | -23.5 |
| miR-211-3p | 476-509 | -23.3 |
| miR-4713-5p | 1760-1780 | -23.3 |
| miR-938 | 1390-1418 | -23.3 |
| miR-942-3p | 513-532 | -23.3 |
| miR-3169 | 929-967 | -22.9 |
| miR-5681b | 1739-1777 | -22.9 |
| miR-4446-5p | 1739-1780 | -22.5 |
| miR-4709-3p | 128-148 | -22.5 |
| miR-4779 | 480-492 | -22.3 |
| miR-4299 | 447-465 | -22.2 |
| miR-4431 | 2086-2102 | -22.2 |
| miR-8087 | 1291-1325 | -22.2 |
| miR-4430 | 2083-2099 | -22.1 |
| miR-544b | 120-151 | -22.1 |
| miR-4657 | 448-481 | -22.0 |
| miR-541-3p | 1593-1627 | -22.0 |
| miR-7150 | 513-530 | -22.0 |
| miR-1244 | 447-466 | -21.8 |
| miR-4434 | 2088-2105 | -21.6 |
| miR-4714-3p | 1340-1362 | -21.6 |
| miR-6129 | 1275-1294 | -21.5 |
| miR-505-5p | 925-944 | -21.4 |
| miR-642a-5p | 1745-769 | -21.4 |
| miR-6511a-3p | 85-88 | -21.4 |
| miR-6770-5p | 117-149 | -21.4 |
| miR-711 | 1314-332 | -21.4 |
| miR-597-5p | 91-94 | -21.3 |
| miR-27b-5p | 925-944 | -21.2 |
| miR-6888-5p | 504-519 | -21.1 |
| miR-7157-5p | 1356-1380 | -21.1 |
| miR-548q | 1954-1973 | -21.0 |
| miR-4421 | 447-464 | -20.9 |
| miR-4435 | 2090-2099 | -20.9 |
| miR-7850-5p | 456-473 | -20.9 |
| miR-624-5p | 1748-1777 | -20.8 |
| miR-3116 | 1009-1024 | -20.7 |
| miR-3936 | 2262-2287 | -20.7 |
| miR-1295b-5p | 85-88 | -20.6 |
| miR-4502 | 997-1015 | -20.6 |
| miR-6503-5p | 1356-1383 | -20.6 |
| miR-6833-3p | 1758-1781 | -20.6 |
| miR-7151-5p | 1739-168 | -20.6 |
| miR-20b-3p | 1684-1705 | -20.5 |
| miR-25-5p | 447-462 | -20.5 |
| miR-4436b-3p | 512-523 | -20.4 |
| miR-4784 | 131-149 | -20.4 |
| miR-525-3p | 1387-1412 | -20.3 |
| miR-642b-3p | 1288-1308 | -20.3 |
| miR-1270 | 447-460 | -20.2 |
| miR-510-5p | 1317-1333 | -20.2 |
| miR-4646-3p | 1747-1769 | -20.1 |
| miR-4429 | 2083-2104 | -20.0 |
| miR-4438 | 2094-2112 | -20.0 |
| miR-4704-5p | 495-512 | -20.0 |
| miR-4753-5p | 504-532 | -19.9 |
| miR-4324 | 1394-1423 | -19.8 |
| miR-4439 | 2133-212 | -19.8 |
| miR-4755-3p | 513-533 | -19.8 |
| miR-5191 | 510-529 | -19.8 |
| miR-6802-3p | 85-90 | -19.8 |
| miR-448 | 1566-112 | -19.7 |
| miR-6507-3p | 1747-1770 | -19.7 |
| miR-135b-5p | 2232-2279 | -19.6 |
| miR-5011-3p | 246-272 | -19.6 |
| miR-5588-5p | 1566-1612 | -19.6 |
| miR-103b | 1005-1023 | -19.3 |
| miR-552-5p | 85-91 | -19.3 |
| miR-8079 | 85-91 | -19.3 |
| miR-3156-3p | 1757-1780 | -19.2 |
| miR-644a | 254-274 | -19.1 |
| miR-3124-3p | 85-90 | -19.0 |
| miR-4436 | 2090-2100 | -19.0 |
| miR-618 | 1757-1777 | -19.0 |
| miR-301b-3p | 106-143 | -18.9 |
| miR-527 | 1561-1608 | -18.9 |
| miR-3165 | 1566-1627 | -18.8 |
| miR-4796-3p | 246-272 | -18.8 |
| miR-885-5p | 85-88 | -18.8 |
| miR-4451 | 246-272 | -18.7 |
| miR-664a-5p | 494-512 | -18.6 |
| let-7g-5p | 127-149 | -18.4 |
| miR-3678-5p | 85-90 | -18.2 |
| miR-4437 | 2090-2109 | -18.2 |
| miR-7162-3p | 128-151 | -18.2 |
| miR-2681-3p | 1647-1663 | -18.0 |
| miR-548b-5p | 462-479 | -18.0 |
| miR-4427 | 1763-1798 | -17.9 |
| miR-4475 | 1288-1297 | -17.8 |
| miR-708-3p | 447-538 | -17.8 |
| miR-590-5p | 184-224 | -17.7 |
| miR-135a-5p | 2246-2279 | -17.6 |
| miR-337-3p | 1560-1780 | -17.6 |
| miR-3657 | 1566-1592 | -17.3 |
| miR-4308 | 1394-1417 | -17.3 |
| miR-4433 | 2088-2095 | -17.2 |
| miR-23c | 1760-1780 | -17.1 |
| miR-3167 | 925-955 | -17.1 |
| miR-1267 | 122-151 | -17 |
| miR-5707 | 1292-1411 | -16.9 |
| miR-4460 | 447-467 | -16.8 |
| miR-3914 | 513-529 | -16.6 |
| miR-6130 | 138-149 | -16.6 |
| miR-1277-5p | 222-245 | -16.5 |
| miR-3924 | 1313-1337 | -16.3 |
| miR-4637 | 1566-1612 | -16.3 |
| miR-4693-3p | 930-946 | -16.1 |
| miR-548an | 467-483 | -16.1 |
| miR-4720-5p | 998-1016 | -16 |
| miR-3973 | 1685-1700 | -15.8 |
| miR-514a-3p | 1680-1696 | -15.7 |
| miR-3115 | 458-476 | -15.6 |
| miR-563 | 1663-1679 | -15.3 |
| miR-4662b | 926-945 | -15 |
| miR-3171 | 259-274 | -14.8 |
| miR-452 | 919-923 | -9.7 |
| miR-3148 | 932-935 | -8.2 |

**Supplementary Table 2**

| **Gene** | **Full name** |
| --- | --- |
| TGFBR2 | transforming growth factor beta receptor 2 |
| GPRIN2 | G protein regulated inducer of neurite outgrowth 2 |
| FAM212B | family with sequence similarity 212, member B |
| NFAM1 | NFAT activating protein with ITAM motif 1 |
| AQP1 | aquaporin 1 |
| FBXO44 | F-box protein 44 |
| STC2 | stanniocalcin 2 |
| PHOSPHO1 | phosphatase, orphan 1 |
| DMBX1 | diencephalon/mesencephalon homeobox 1 |
| DYNLL2 | dynein, light chain, LC8-type 2 |
| AVPI1 | arginine vasopressin-induced 1 |
| BCL2L2-PABPN1 | BCL2L2-PABPN1 readthrough |
| AKIRIN1 | akirin 1 |
| BAZ2A | bromodomain adjacent to zinc finger domain, 2A |
| LARP4B | La ribonucleoprotein domain family, member 4B |
| NRF1 | nuclear respiratory factor 1 |
| PABPN1 | poly(A) binding protein, nuclear 1 |
| RPS9 | ribosomal protein S9 |
| CYP4F11 | cytochrome P450, family 4, subfamily F, polypeptide 11 |
| ELAVL3 | ELAV like neuron-specific RNA binding protein 3 |
| GPATCH2L | G patch domain containing 2-like |
| GOLGA7B | golgin A7 family, member B |
| CTH | cystathionase (cystathionine gamma-lyase) |
| ZBTB4 | zinc finger and BTB domain containing 4 |
| ARL10 | ADP-ribosylation factor-like 10 |
| SMC1A | structural maintenance of chromosomes 1A |
| AGO1 | argonaute RISC catalytic component 1 |
| CHD8 | chromodomain helicase DNA binding protein 8 |
| OTUD7B | OTU domain containing 7B |
| NAIF1 | nuclear apoptosis inducing factor 1 |
| RCAN1 | regulator of calcineurin 1 |
| SLC39A9 | solute carrier family 39, member 9 |
| LDB2 | LIM domain binding 2 |
| SLC6A4 | solute carrier family 6 (neurotransmitter transporter), member 4 |
| LRRC59 | leucine rich repeat containing 59 |
| FAM57B | family with sequence similarity 57, member B |
| MIF4GD | MIF4G domain containing |
| CBX6 | chromobox homolog 6 |
| ZDHHC3 | zinc finger, DHHC-type containing 3 |
| HMGCS1 | 3-hydroxy-3-methylglutaryl-CoA synthase 1 (soluble) |
| GABBR2 | gamma-aminobutyric acid (GABA) B receptor, 2 |
| TMED8 | transmembrane emp24 protein transport domain containing 8 |
| EPHA10 | EPH receptor A10 |
| SRP19 | signal recognition particle 19kDa |
| STOML1 | stomatin (EPB72)-like 1 |
| CAB39L | calcium binding protein 39-like |
| MAFG | v-maf avian musculoaponeurotic fibrosarcoma oncogene homolog G |
| ERF | Ets2 repressor factor |
| RELT | RELT tumor necrosis factor receptor |
| MAPK6 | mitogen-activated protein kinase 6 |
| PPP5C | protein phosphatase 5, catalytic subunit |
| UBFD1 | ubiquitin family domain containing 1 |
| BTN2A2 | butyrophilin, subfamily 2, member A2 |
| ATP1A2 | ATPase, Na+/K+ transporting, alpha 2 polypeptide |
| KLHL3 | kelch-like family member 3 |
| FBXL19 | F-box and leucine-rich repeat protein 19 |
| NHP2 | NHP2 ribonucleoprotein |
| WSCD2 | WSC domain containing 2 |
| ZNF710 | zinc finger protein 710 |
| GPR37L1 | G protein-coupled receptor 37 like 1 |
| HIPK1 | homeodomain interacting protein kinase 1 |
| EFNB1 | ephrin-B1 |
| BTN2A1 | butyrophilin, subfamily 2, member A1 |
| METTL14 | methyltransferase like 14 |
| FRRS1L | ferric-chelate reductase 1-like |
| MEFV | Mediterranean fever |
| NDRG2 | NDRG family member 2 |
| TERT | telomerase reverse transcriptase |
| UBTF | upstream binding transcription factor, RNA polymerase I |
| CBX5 | chromobox homolog 5 |
| BCL2L2 | BCL2-like 2 |
| NPAS4 | neuronal PAS domain protein 4 |
| CCND3 | cyclin D3 |
| GRINA | glutamate receptor, ionotropic, N-methyl D-aspartate-associated protein 1 (glutamate binding) |
| TCF4 | transcription factor 4 |
| APC2 | adenomatosis polyposis coli 2 |
| INSM2 | insulinoma-associated 2 |
| GGCX | gamma-glutamyl carboxylase |
| STMN4 | stathmin-like 4 |
| ENSA | endosulfine alpha |
| ACTR3 | ARP3 actin-related protein 3 homolog (yeast) |
| CEP170B | centrosomal protein 170B |
| CHD7 | chromodomain helicase DNA binding protein 7 |
| RAP1GAP2 | RAP1 GTPase activating protein 2 |
| BCL11B | B-cell CLL/lymphoma 11B (zinc finger protein) |
| TSPAN9 | tetraspanin 9 |
| BRD4 | bromodomain containing 4 |
| CYP26B1 | cytochrome P450, family 26, subfamily B, polypeptide 1 |
| DIAPH1 | diaphanous-related formin 1 |
| PGM2L1 | phosphoglucomutase 2-like 1 |
| EBF4 | early B-cell factor 4 |
| TNPO1 | transportin 1 |
| WIPF2 | WAS/WASL interacting protein family, member 2 |
| WASF2 | WAS protein family, member 2 |
| PDE7A | phosphodiesterase 7A |
| GAB2 | GRB2-associated binding protein 2 |
| SMG6 | SMG6 nonsense mediated mRNA decay factor |
| IVD | isovaleryl-CoA dehydrogenase |
| CD177 | CD177 molecule |
| B3GNT7 | UDP-GlcNAc:betaGal beta-1,3-N-acetylglucosaminyltransferase 7 |
| RPRD2 | regulation of nuclear pre-mRNA domain containing 2 |
| SLC29A2 | solute carrier family 29 (equilibrative nucleoside transporter), member 2 |
| GNL1 | guanine nucleotide binding protein-like 1 |
| PRPF40A | PRP40 pre-mRNA processing factor 40 homolog A (S. cerevisiae) |
| CNOT6L | CCR4-NOT transcription complex, subunit 6-like |
| FAM160B2 | family with sequence similarity 160, member B2 |
| RBBP5 | retinoblastoma binding protein 5 |
| KIAA1549 | KIAA1549 |
| SNX11 | sorting nexin 11 |
| KCND3 | potassium voltage-gated channel, Shal-related subfamily, member 3 |
| PHLDA3 | pleckstrin homology-like domain, family A, member 3 |
| SLC39A13 | solute carrier family 39 (zinc transporter), member 13 |
| HCN4 | hyperpolarization activated cyclic nucleotide-gated potassium channel 4 |
| BCL7A | B-cell CLL/lymphoma 7A |
| ATG9A | autophagy related 9A |
| COLGALT2 | collagen beta(1-O)galactosyltransferase 2 |
| GDAP1L1 | ganglioside induced differentiation associated protein 1-like 1 |
| DNMT3A | DNA (cytosine-5-)-methyltransferase 3 alpha |
| RIMKLA | ribosomal modification protein rimK-like family member A |
| NFIC | nuclear factor I/C (CCAAT-binding transcription factor) |
| KMT2D | lysine (K)-specific methyltransferase 2D |
| TMED1 | transmembrane emp24 protein transport domain containing 1 |
| PRICKLE2 | prickle homolog 2 (Drosophila) |
| PTAFR | platelet-activating factor receptor |
| SMIM2 | small integral membrane protein 2 |
| ETNK2 | ethanolamine kinase 2 |
| LEMD2 | LEM domain containing 2 |
| GTPBP1 | GTP binding protein 1 |
| CISD3 | CDGSH iron sulfur domain 3 |
| BCL9L | B-cell CLL/lymphoma 9-like |
| FMNL3 | formin-like 3 |
| TK2 | thymidine kinase 2, mitochondrial |
| ITPA | inosine triphosphatase (nucleoside triphosphate pyrophosphatase) |
| KIAA0141 | KIAA0141 |
| KIAA1614 | KIAA1614 |
| MTA3 | metastasis associated 1 family, member 3 |
| CNP | 2',3'-cyclic nucleotide 3' phosphodiesterase |
| FCHSD1 | FCH and double SH3 domains 1 |
| TFCP2L1 | transcription factor CP2-like 1 |
| DCUN1D3 | DCN1, defective in cullin neddylation 1, domain containing 3 |
| MBD2 | methyl-CpG binding domain protein 2 |
| PIP4K2B | phosphatidylinositol-5-phosphate 4-kinase, type II, beta |
| MAP4K2 | mitogen-activated protein kinase kinase kinase kinase 2 |
| AQP1 | aquaporin 1 |
| MOBP | myelin-associated oligodendrocyte basic protein |
| KCNK10 | potassium channel, subfamily K, member 10 |
| BTN3A2 | butyrophilin, subfamily 3, member A2 |
| ITIH5 | inter-alpha-trypsin inhibitor heavy chain family, member 5 |
| BTN3A1 | butyrophilin, subfamily 3, member A1 |
| ZNF652 | zinc finger protein 652 |

**Supplementary Table 3**

| **Name** | **Sequence (5’ to 3’)** |
| --- | --- |
| Mimics NC | UUGUACUACACAAAAGUACUG |
| MiR-1306 | CCACCUCCCCUGCAAACGUCCA |
| Inhibitor NC | CAGUACUUUUGUGUAGUACAA |
| MiR-1306 inhibitor | UGGACGUUUGCAGGGGAGGUGG |
| NC-siRNA | UUCUCCGAACGUGUCACGUTT |
|  | ACGUGACACGUUCGGAGAATT |
| TGFBR2-siRNA | GGAGGAAGAAUGACGAGAATT |
|  | UGUGGUUGAUGUUGUUGGCTT |
| SMAD4-siRNA | CACCAGGAAUUGAUCUCUCAGGAUU |
|  | AAUCCUGAGAGAUCAAUUCCUGGUG |

**Supplementary Table 4**

| **Genes** | **Primer sequence (5’ to 3’)** | **Usage** |
| --- | --- | --- |
| miR-1306 | CCTGTTGTCTCCAGCCACAAAAGAGCACAATATTTCAGGAGACAACAGGTGGACGT | Reverse-transcription |
| miR-1306 | F: CGGGCCCACCTCCCCTGCAA | qPCR |
|  | R: CAGCCACAAAAGAGCACAAT |  |
| TGFBR2 | F: TGGCTCCTGAAGTCCTAGAGT | qPCR |
|  | R: GAACCAAAGGGTGGCTCAT |  |
| DGCR8 | F: GTATGCAGTGCTGGATGAGTTAGA | qPCR |
|  | R: CTTCCAGCAGAGCATCAACATC |  |
| U6 | F: GCTTCGGCAGCACATATACT | qPCR |
|  | R: TTCACGAATTTGCGTGTCAT |  |
| GAPDH | F: CGTGCGGTTGTGGATCT | qPCR |
|  | R: CTCAGTGTAGCCCAGGAT |  |

**Supplementary Table 5**

| **Plasmids** | **Primer sequence (5’ to 3’)** | **Usage** |  |
| --- | --- | --- | --- |
| prom-408 | | F: CGAGCTCCTGGGTCTCGACGGACTT | Promoter vector  construction |
|  | | R: CTAGCTAGCGCCACTACAGTTCGGGTCTA |  |
| prom-877 | | F: CGAGCTCGGATGCTGTGGTGAAGTTTAGT | Promoter vector  construction |
|  | | R: CTAGCTAGCGCCACTACAGTTCGGGTCTA |  |
| prom-1300 | | F: CGAGCTC CTCAAGTAGGGACTGAATGGT | Promoter vector  construction |
|  | | R: CTAGCTAGCGCCACTACAGTTCGGGTCTA |  |
| prom-1631 | | F: CGAGCTCACCCTCTTCTTGGTCAGTGTT | Promoter vector  construction |
|  | | R: CTAGCTAGCGCCACTACAGTTCGGGTCTA |  |
| pGL3–mut1 | | F: GGAGGAAGCCAGGTGCCCCTCCGGCATGAAGACAG | Mutation |
|  | | R: GGCACCTGGCTTCCTCCACCTGCGAAGAGACGTAA |  |
| pGL3–mut2 | | F: CATGAAGAACGACTCGCTTAGTCGTCAGTCATTTA | Mutation |
|  | | R: AGCGAGTCGTTCTTCATGCCGGAGGGGCAC |  |
| pGL3–mut3 | | F: CTATGGAGCCGTGTGAGAGCCCCTGTCCTCTCCCG | Mutation |
|  | | R: CTCACACGGCTCCATAGTAAAAGTGCTTAAGACTA |  |

**Supplementary Table 6**

| **Name** | **Primer sequence (5’ to 3’)** | **Product Length (bp)** |
| --- | --- | --- |
| Site X | P1: CAGCAAAGGGTGGCAAGGT | 145 |
|  | P2: ATGCCTGTGCCACTTTCAACT |  |
| SBE1 | F: GTTCTGACCACATGTTAGTAGCGC | 123 |
|  | R: GTCTTCATGCCGGAGGGG |  |
| SBE2/3 | F: GCCCCTCCGGCATGAAGA | 187 |
|  | R: AACATGCACAAGTCCGTCGAGA |  |
| SBE4 | F: CGGACTTGTGCATGTTAGCTGTG | 187 |
|  | R: TGGGTTCTCCATGACCGCTC |  |
